# Deep Learning for Structural Health Monitoring: Data, Algorithms, Applications, Challenges, and Trends

**DOI:** 10.3390/s23218824

**Published:** 2023-10-30

**Authors:** Jing Jia, Ying Li

**Affiliations:** Department of Civil Engineering, College of Engineering, Ocean University of China, Qingdao 266100, China; liying4555@stu.ouc.edu.cn

**Keywords:** structural health monitoring, deep learning algorithms, damage detection, data acquisition, facilities

## Abstract

Environmental effects may lead to cracking, stiffness loss, brace damage, and other damages in bridges, frame structures, buildings, etc. Structural Health Monitoring (SHM) technology could prevent catastrophic events by detecting damage early. In recent years, Deep Learning (DL) has developed rapidly and has been applied to SHM to detect, localize, and evaluate diverse damages through efficient feature extraction. This paper analyzes 337 articles through a systematic literature review to investigate the application of DL for SHM in the operation and maintenance phase of facilities from three perspectives: data, DL algorithms, and applications. Firstly, the data types in SHM and the corresponding collection methods are summarized and analyzed. The most common data types are vibration signals and images, accounting for 80% of the literature studied. Secondly, the popular DL algorithm types and application areas are reviewed, of which CNN accounts for 60%. Then, this article carefully analyzes the specific functions of DL application for SHM based on the facility’s characteristics. The most scrutinized study focused on cracks, accounting for 30 percent of research papers. Finally, challenges and trends in applying DL for SHM are discussed. Among the trends, the Structural Health Monitoring Digital Twin (SHMDT) model framework is suggested in response to the trend of strong coupling between SHM technology and Digital Twin (DT), which can advance the digitalization, visualization, and intelligent management of SHM.

## 1. Introduction

Various damages may occur in building structures after long terms of environmental loads such as wind, earthquake, automobile, environmental vibration, etc. It could consequently impact the building’s general stability and safety and lead to serious loss of life and property [1]. For this reason, SHM is critical to the facility, whether it is the whole facility or key components of the facility. For example, a healthy concrete structure needs to maintain high strength and good durability, which is closely related to the material and ratio of mortar [2,3]. The reduction of strength and durability reflects the insecurity of the structure, which requires SHM to evaluate the durability through vibration, stress, and other parameters.

Housner et al. [4] defined SHM as using sensing technology and structural characterization to detect changes that may indicate damage or degradation. Dong et al. [5] proposed that SHM is the process of tracking operational status, assessing the condition, and detecting various types of structural damage. In summary, the primary purpose of SHM is to enable damage detection and condition assessment by sensing, recognizing, and evaluating structural operating conditions. Its core component is Damage Detection (DD), i.e., structural damage identification, localization, and assessment. Damage detection was separated into four categories in the Rytter study [6]. (1) Detection: clarification of the presence of damage. (2) Location: determination of the location and coordinates of the damage. (3) Assessment: measurement of the severity of the damage. (4) Consequence: acquisition of the actual safety information of the structure in the determined damage state. There are four critical elements of SHM: data acquisition, system identification, condition assessment, and maintenance [7]. Among them, the sensor and sensor data in the data acquisition stage are the core of SHM applications. The operational status data of the structure is obtained through contact (accelerometers, strain gauges, fiber optic sensors) and non-contact sensors (high-speed cameras, drones, smartphones). Based on the data, appropriate data processing methods (Machine Learning (ML), DL, signal processing methods) are adopted to select damage-sensitive features for damage identification and condition assessment. Finally, according to the evaluation results, necessary measures are taken to maintain the safety and service life of the structure.

Early SHM relied on visual inspection, which had the drawbacks of low efficiency, poor accuracy, susceptibility to subjective factors, and high cost of labor and time [8]. Gradually, Non-Destructive Testing (NDT) methods, such as Acoustic Emission (AE) [9], ultrasonic [10], X-ray [11], magnetic particle inspection [12], eddy current [13], and other detection methods, are frequently utilized for monitoring the key component of the structure [13]. Currently, the most commonly used SHM methods are vibration-based SHM [14,15,16,17,18], AE-based SHM [9,19,20], Guided Wave (GW) based SHM [21,22,23,24,25], and Electro-Mechanical Impedance (EMI) based SHM [26,27]. Among them, vibration-based SHM is the most popular one by analyzing the relationship between vibration characteristics and damage states. In general, DD methods for SHM can be divided into model-driven methods and data-driven methods [28]. The former uses a Finite Element Model (FEM) calibrated by sensor data and optimization algorithms to analyze structural damage. The latter uses sensor data directly to detect structural damage. Moreover, the data-driven methods, which directly obtain damage features from sensor data to estimate the structural state, are more flexible and have attracted more and more attention. Thereby, the Gaussian model, ML, and DL have emerged as popular data analysis methods.

DL is a sub-field of ML in artificial intelligence, which has become an important research topic with the development of big data, computer software, and hardware. DL comes from the research of Artificial Neural Networks (ANN), which learn the characteristics of data samples by imitating the structure of human brain neurons and then give the interpretation of data. As a multi-layer neural network, DL will feed the features of the original signal detected from the low to the high layer, which is more conducive to learning advanced features. Compared with traditional ML, DL is capable of automatic feature extraction and is suitable for processing other high-dimensional data such as images, video, and audio. Therefore, DL is used in various fields, such as speech recognition [29,30], machine translation [31,32], image classification [33,34], etc. In addition, DL has also been applied to SHM to identify, locate, and evaluate various damages by extracting advanced damage features from data such as pictures and vibration signals [15,35,36].

In the field of SHM, DL methods can be divided into image-based and vibration-based methods. The former uses the DL algorithm to extract features from the structural surface damage images and realizes image processing through classification, localization, and segmentation. First, the images are classified and labeled as damaged or undamaged. Secondly, the damaged area and damaged coordinates are located and identified. Finally, pixel-level segmentation marks image pixels as damaged or undamaged [37]. The mainstream DL algorithm applied to structural surface image damage detection is mainly a Convolutional Neural Network (CNN), which can realize image classification, object recognition, and semantic segmentation. The CNN algorithm involved includes Two-Dimensional Convolutional Neural Networks (2D-CNN), You Look Only Once (YOLO), U-Net, Region-based Convolutional Neural Networks (R-CNN), Fully Convolutional Networks (FCN), Mask Region-Based Convolutional Neural Networks (Mask R-CNN), etc. Li et al. [38] used the sliding window method to cover the image of the steel bridge and used convolution and pooling methods in CNN to classify the steel bridge’s bolts, nuts, and nut holes. Kao et al. [36] used YOLOv4 to identify bridge cracks through boundary frame selection and extracted crack contours using the edge detection method to achieve crack quantification. Panta et al. [39] proposed an iterative loop U-Net to segment crack images of levees into crack pixels and non-crack pixels to identify crack shapes accurately. Therefore, the DL algorithm can make use of structural surface defect images of bridges, frame structures, dams, tunnels, and other infrastructures for crack detection [40,41,42,43,44], bolt loosening detection [45,46,47], rebar surface defect detection [48], delamination and reinforcement exposure detection [49], displacement detection [50,51,52,53], voids detection [54], etc. The primary sensors used to acquire structural surface defect images mainly include high-resolution cameras, infrared and near-infrared cameras, mobile phones, radar systems, and so on [55]. Unmanned Air Vehicles (UAVs) can carry various sensors, hover, and fly through remote sensing technology to obtain high-definition damage images.

Image-based SHM cannot detect internal damage, while vibration-based SHM can extract natural frequencies, vibration modes, and damping ratios from vibration data to detect internal damage. However, when processing vibration signals using 2D-CNN, it is necessary to convert one-dimensional data into two-dimensional data. On this basis, One-Dimensional Convolutional Neural Networks (1D-CNN) with simple architecture and low computational complexity are applied to SHM to directly process 1D data for crack detection [56,57], corrosion detection [58], multi-type damage identification [1], abnormal data detection [59,60], etc. In addition to CNN, Recurrent Neural Networks (RNN) can identify the time features of data. Through Long Short-Term Memory (LSTM) and Gated Recurrent Unit (GRU), it can not only realize multi-type damage identification and location [61], crack detection [62,63], and dam displacement monitoring [64,65,66] but also detect data anomalies caused by sensor faults or environmental changes [67,68,69], and even predict missing data [70,71,72,73]. There are also unsupervised learning algorithms, such as auto-encoder and Generative Adversarial Networks (GAN), which directly use unlabeled data for feature extraction. They can be applied for dimensionality reduction [74,75], image generation [43,76], lost data reconstruction [77,78,79], AE source localization [80,81], crack detection [82,83,84], and so on.

Twenty-three literature reviews have been retrieved with the theme of DL applied to SHM (see Section 2 for the specific retrieval process) and analyzed from three levels. At the data level, there are two reviews for vibration and image data [55,85] and two for specific sensors [86,87]. At the algorithm level, two papers target CNN [37,88], two artificial intelligence [89,90], and one proposes a new machine learning paradigm [91]. At the application level, there are seven papers on specific structures, including bridges [92,93,94,95], wind turbine blades [96], composite structures [97], and threaded fasteners [98]. This is followed by three papers on cracking [99,100,101], one on data science [102], and three on the construction industry [35,103,104]. Given this, this paper will be the first comprehensive review of the application of DL to SHM from three perspectives: data, algorithm, and application. Consequently, this review can be a reference for related researchers in both academia and industry. The main concerns of the article are listed below.

- At the data level, we studied the data types adopted by DL for SHM and compared their scope of application. They were followed by analysis and comparison of the different data acquisition methods.
- At the algorithm level, we analyzed the DL algorithm types commonly used in SHM and clarified the data types, core functions, and applications of different algorithms.
- At the application level, we summarized the popular application objects and application functions of DL for SHM on different facilities and facility components.
- Challenges and trends were presented at three levels: data, algorithm, and application. Moreover, combined with the structural model data, the SHMDT framework is constructed to develop the SHM in the direction of digitization and intelligence.

This paper is divided into seven sections. Section 1 sums up the SHM methodology and the application of DL in SHM. Section 2 puts forward the research methodology of the paper. Next, the analysis is carried out according to three dimensions: data, algorithms, and applications, with corresponding Section 3, Section 4 and Section 5. Finally, Section 6 explores the current challenges and future trends and puts forward the SHMDT model framework by combining SHM with the current research hotspot DT.

## 2. Research Methodology

**Figure 1:**
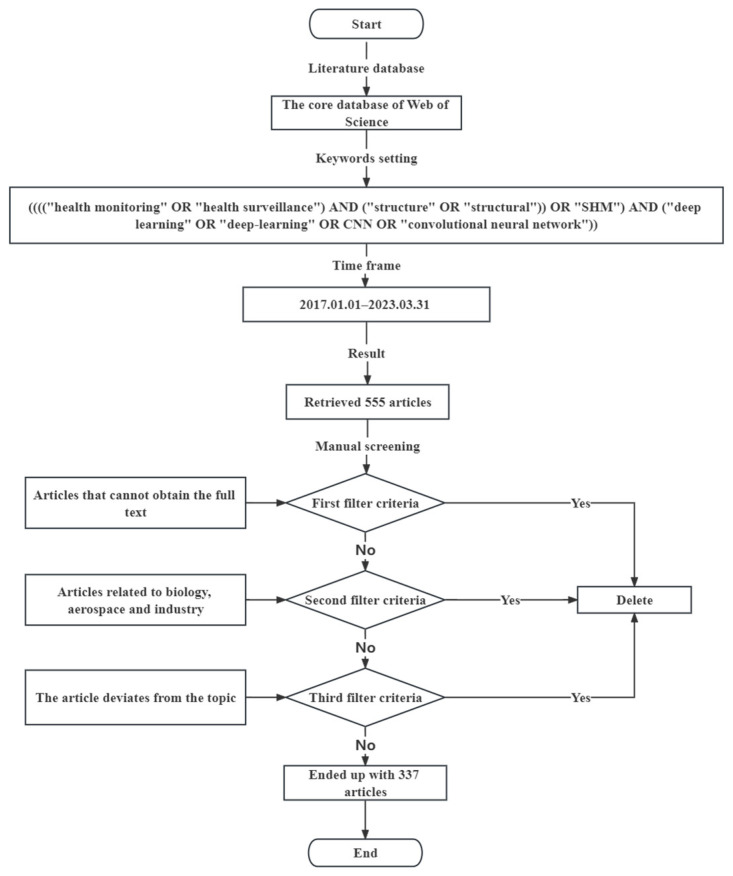
Literature retrieval flow chart.

In this paper, 337 articles were identified for review following the process in Figure 1. The customized selection process is as follows:

(1) Literature database: Web of Science was chosen as the search database.
(2) Keywords setting: Considering that CNN is the most commonly applied algorithm in DL, CNN was also selected as a keyword in addition to “SHM” and “DL.” The selected keywords and their parallel relationships were: ((((“health monitoring” OR “health surveillance”) AND (“structure” OR “structural”)) OR “SHM”) AND (“deep learning” OR “deep-learning” OR CNN OR “convolutional neural network”)).
(3) Time frame: To study the latest progress of SHM, we selected the relevant literature in the past six years, and the corresponding search time range was 1 January 2017–31 March 2023..
(4) Result: Through retrieval, a total of 555 journal articles in related fields were obtained.
(5) Manual screening: Manual screening includes preliminary screening and rescreening. The preliminary screening based on the title and abstract of the article can exclude articles related to biology, aerospace, and industry, as well as articles where we cannot obtain the full text. Rescreening was performed by reading all articles in detail to exclude articles with topic deviations, and the final number of articles was 337, including 23 review articles.

**Figure 2:**
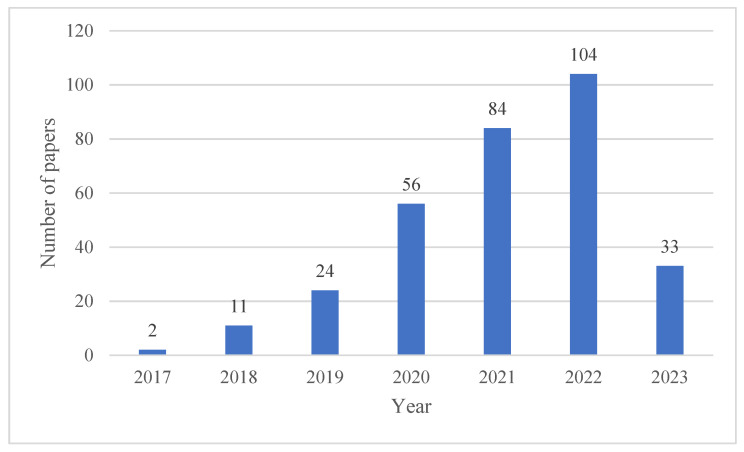
Number of DL and SHM-related articles from 2017 to 2023.

**Figure 3:**
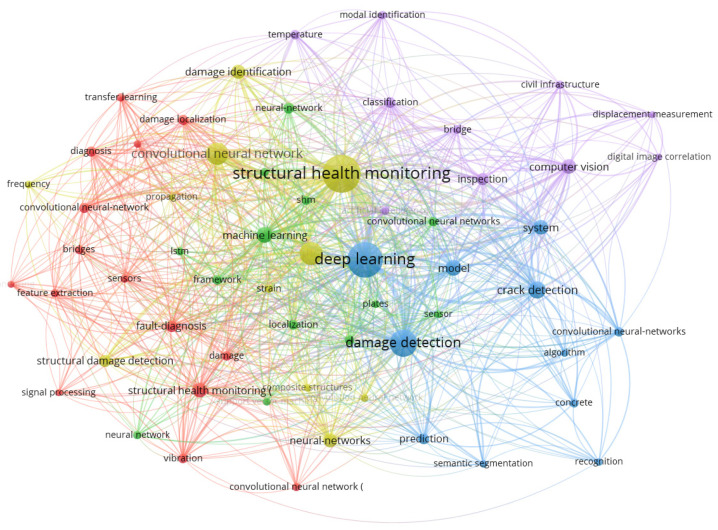
Keyword co-occurrence graph.

A preliminary analysis of the retrieved articles in years is shown in Figure 2 from 2017 to 2023. This figure shows an increasing trend, indicating continuous attention. Figure 3 is the keyword co-occurrence graph generated by VOSviewer, which reflects the number and distribution of keywords in the filtered articles. The graph shows the top 60 keywords with the highest number of occurrences, where the larger the node and font size, the higher the number of occurrences. It shows that hot research topics include structural health monitoring, deep learning, damage detection, convolutional neural networks, and crack detection.

## 3. Data Types

**Figure 4:**
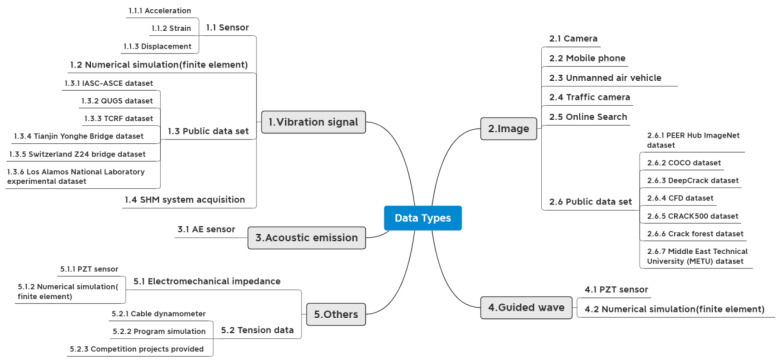
Data types summary diagram.

Figure 4 shows four commonly used data types in SHM: vibration signals, images, AE signals, and GW signals. Vibration data is one of the most frequently used signals, mainly recorded by vibration sensors installed on the structure. Images containing damage, such as cracks, corrosion, peeling, etc., are another common type of data, mainly obtained through devices such as cameras and smartphones. In addition, there are AE and GW signals collected by specific sensors, which can be used to achieve DD by using characteristics such as waveforms. Detailed information about the data and how it was acquired is shown in Table S1.

### 3.1. Vibration Signal

Vibration-based SHM is a method to identify structural damage’s existence, location, and severity by analyzing the correlation between the vibration characteristics of vibration signals (such as acceleration, displacement, and strain) and the state of structural damage [8]. The main reason is that structural damage can cause changes in physical properties and affect vibration characteristics. Vibration-based SHM can be divided into parametric and non-parametric methods. The former detects damage by comparing parameters related to the physical properties of damaged and undamaged structures [105], such as modal frequency, modal mass, modal damping, stiffness, and modal shape [106,107]. The latter directly extracts damage-sensitive features from the original vibration signal and then uses the classifier to evaluate the structure’s health. In contrast to parametric methods, the accuracy of non-parametric methods is independent of specific modes but requires powerful data processing tools such as DL and ML [108]. In the studied literature, vibration-based SHM is mainly used in crack [109], brace damage [110,111,112], bolt loosening [113,114], and stiffness reduction [115,116] detection.

Vibration signals are usually obtained by sensors covering the structure. Still, considering the operability and cost, receiving sufficient vibration signals with different structural damage conditions takes much work. Constructing numerical models, especially FEM, can solve the problem of insufficient data. The FEM can be updated using optimization algorithms to minimize the simulated and experimental data gap. Wang et al. [117] constructed the FEM of a steel frame and updated the model to generate a dataset including vibration mode and stiffness, which was used to train and test the ResNet model, and realized the crack detection of a steel frame. Wang et al. [118] proposed a damage identification method for cable dome structures combining DT and layered DL. They realized damage type identification by generating damage data through FE analysis, with an accuracy rate of 88.317%. In addition, there are some public datasets available for research. For example, the IASC-ASCE benchmark dataset [110,113,119,120,121,122], Three-span Continuous Rigid Frame Bridge (TCRF) dataset [109,122,123], Qatar University Grandstand Simulator (QUGS) [82,110,114,124], Los Alamos National Laboratory experimental dataset [114,125], Tianjin Yonghe Bridge dataset [82,121,124], Switzerland Z24 bridge dataset [1], etc. With consent, it is possible to use data from other people’s articles [126,127,128]. Finally, the SHM system also acquires data through sensors. Ni et al. [129] used the data from the SHM system of a long-span bridge in China. The SHM system has more than 170 sensors, including accelerometers, strain sensors, displacement sensors, etc., which can collect data for monitoring structural conditions.

### 3.2. Image

Image-based SHM can obtain damage-sensitive features from images of DD. Damage-sensitive feature extraction can be achieved through image processing techniques, ML, and DL [100], where DL can automatically extract features for image classification, object recognition, and semantic segmentation tasks. DL-based SHM mainly uses structural damage images to classify or segment surface defects to achieve crack detection [130,131,132], bolt loosening detection [47], steel bar exposure detection [49,133], or vehicle recognition through vehicle images captured by road cameras [134,135].

The most direct means of acquiring images is through cameras. The resolution of the consumer-grade digital camera is gradually improved and can be used to collect damaged photos. Xu et al. [136] used a measurement system consisting of a digital camera, LED lighting, and a mobile vehicle to collect images of the tunnel interior. The collected images were then used for detecting the cracks through Mask R-CNN. With the improvement in shooting performance of smartphones, it is a convenient and low-cost way to use mobile phones as image acquisition devices. Furthermore, high-mobility UAVs are famous for capturing images of essential parts that are difficult for humans to reach. Kang et al. [137] input video data collected by autonomous UAV into CNN, which used sliding window technology to detect and locate concrete cracks. Two types of image resources are directly and publicly available. One is the image that can be searched on search engines such as Google, and the other is a variety of image datasets. For example, PEER Hub ImageNet [138], COCO dataset [139,140], Middle East Technical University (METU) dataset [141,142], DeepCrack [143], CFD dataset [143], CRACK500 [143], Crack forest dataset [144], etc. Finally, Jang et al. [145] utilized a visual camera and an infrared camera to collect hybrid images and then input them into GoogLeNet, which can improve the ability to detect cracks. Chen et al. [76] used a GAN model to generate virtual crack images for training a CNN classifier and then verified the good performance using authentic images. Lu et al. [47] synthesized the bolt dataset using Unreal Engine 5 and then used YOLOv7 to effectively detect the bolt loosening angle. The use of synthetic datasets can improve the efficiency of database building, reduce the cost, and improve the training performance of the model.

### 3.3. Acoustic Emission

Acoustic emission is the radiation of sound waves in solids when the structure changes reversibly or irreversibly [146]. AE-based SHM is a passive, non-destructive testing method for analyzing structural damage characteristics after converting AE signals into electrical waveforms [19]. The AE signal does not require external excitation and is generated spontaneously as damage appears and expands, thus allowing continuous monitoring. In the laboratory, pencil lead break and impact are often used as artificial AE sources, and then AE source location and defect characterization can be realized through DL. The main applications are fatigue crack localization in metal plates [80,81,147], damage localization in composite panels [19], and concrete crack detection [62].

Acquiring AE signals relies heavily on AE sensors, and FE simulations can also obtain AE signals. Yang et al. [147] simulated the artificial AE source of the crack through the pencil lead break experiment on the Q235 B steel plate. The signal collected by the sensor forms a dataset, which was used to train the stacked denoising auto-encoder to determine the coordinates of the AE source. Chen et al. [146] collected AE signals through AE monitoring systems installed on railway tracks, which contained more noise than signals captured in the laboratory. Then, the DL model is trained by transfer learning combined with laboratory data, and the process of the rail from intact to rupture is wholly inferred. Garrett et al. [148] found that the FEM-simulated AE signals matched the experimentally collected AE signals, implying the accuracy of the FE model parameters. The collected signal was input into a CNN to classify and predict different crack lengths automatically.

### 3.4. Guided Wave

Ultrasonic Guided Wave (UGW) is a non-destructive testing technique applied to detect internal damage in structures. GW is formed by the complex reaction of ultrasound waves in a medium through multiple reflections and interference. Commonly used GWs include Rayleigh and Lamb waves, which can be scanned over a large area and are sensitive to minor damage [149]. When using the GW-based SHM method, the GW signal is employed to analyze damage characteristics for damage localization and damage assessment, e.g., to assess the state of crack extension [21], to locate and evaluate damage in aluminum plates [65,149], and to monitor stresses [24]. However, the multimodal and dispersive nature of GW increases the complexity of the analysis and can be dealt with by time or frequency domain methods, ML, and DL methods. DL is a more suitable processing tool than the other two methods, thanks to its robust feature extraction and fusion capabilities. It can present the shape and location of the damage by direct GW imaging, where the key is to obtain GW imaging features.

The Piezoelectric Transducer (PZT) can be flexibly arranged on the subject to form a rational array, with one part used for transmitting GW signals and the other for receiving GW signals. Chen et al. [21] used two PZT sensors to collect the GW signal of the original state of the aluminum plate and the GW signal during the gradual generation of fatigue cracks. The GW signal was then converted into a time-frequency map by wavelet transform and fed into the CNN to evaluate the length of the crack. Similarly, the GW signal can also be simulated by FE as a supplement and substitute for the experimental data. Pandey et al. [149] adopted both FE simulations and PZT sensors to acquire lamb wave signals and input the one-dimensional signals directly into a 1D-CNN to detect the presence and location of the damage. In addition, lasers and ultrasonic probes [150] can be utilized as GW transmission and reception devices. Finally, GW data can be acquired through the Open Guided Waves project [10].

### 3.5. Others

EMI-based SHM uses low-cost, lightweight PZT sensors to stimulate structures and then collects EMI signals to identify localized damage to metallic structures. de Oliveira et al. [151] used PZT patches to capture EMI signals for three damage conditions in aluminum plates and then converted the signals into RGB frames that were applied to train a CNN for damage identification. Chen et al. [152] used FE to simulate resistance tomography data and an ANN to predict the cracking patterns of reinforced concrete members.

Ground Penetrating Radar (GPR) sensors can acquire GPR data. Ahmed et al. [153] developed an automated bridge reinforcement detection and position system, which used ResNet and Kmeans to process GPR data. In order to check the cable damage condition of the cable-stayed bridge, it is necessary to clarify the cable tension, and the cable tension meter and practical advanced analysis program simulation software can help. According to concrete construction records in the Yamaguchi Prefecture, Hosoda et al. [154] proposed a method to predict the maximum crack width by inputting data such as reinforcement ratio, concrete material information, and ambient temperature into the artificial neural network. In addition, other data, such as impulse signals, temperature, and wind speed, are auxiliary data required to study specific damage situations that can be obtained using the appropriate sensors or other feasible means.

### 3.6. Summary

**Figure 5:**
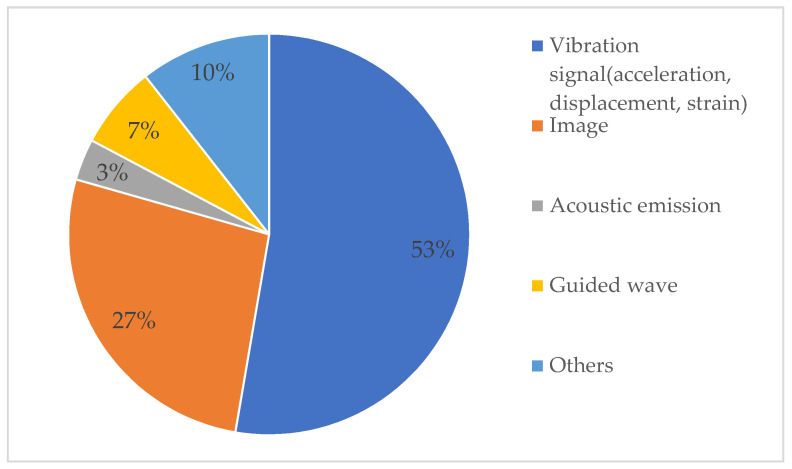
Data types percentage diagram.

Based on categorizing the research literature at the data level in Supplementary Table S1, we obtained the data type percentages in Figure 5. The ratio can reflect the popularity of different data types and support further research. We must clear the research objects and conditions before selecting data and collection methods to increase cost-effectiveness, operational feasibility, and test result accuracy. Table 1 shows the comparison of different data types. It can be found that vibration-based SHM is suitable for various types of damage, such as cracks, brace damages, stiffness reduction, etc. During data collection, attention should be paid to sensors and noise. Because the sensor will be damaged due to environmental changes or power problems, resulting in data loss. Also, the collected data can be contaminated by random noise, which affects the feature extraction efficiency, thus reducing DD’s accuracy. Stronger generalization capabilities of the model are necessary due to the time-varying properties of the vibration signals during processing. According to the study, the method has high detection accuracy but is insensitive to minor injuries. Image-based SHM only deals with surface damage, such as cracks, corrosion, spalling, etc. The influence of the shooting equipment and shooting environment on the quality of photos is a problem to be considered in the data process. Data collection must take environmental factors like illumination and the capabilities of the shooting equipment into account. Compared with other methods, this method processes images through DL, which requires high time complexity and strong data storage capacity. Accordingly, it is possible to achieve damage visualization and increase detection accuracy. AE-based SHM is mostly applied to localize artificial AE sources in laboratory environments, mainly because AE signals in real environments are weak and uncertain but strongly noisy. GW-based SHM is primarily used for metal structure DD and is characterized by remote monitoring, extensive area scanning, and high sensitivity. The sensor and noise factors also impact its data collection process. Processing data can be challenging because GW signals are multi-modal and discrete.

Table 2 summarizes the comparison of different data acquisition methods. Three considerations, including time spent, cost, and data accuracy, must be considered while choosing the appropriate data acquisition method. Firstly, it typically takes a long time to gather enough data when employing sensors, which could be months or years. The price of sensors is relatively low, but the quantity is positively correlated to the size of the facility. With this method, data collection is highly accurate. The FE method allows data to be generated from the model with moderate data accuracy. In this process, FEM requires detailed modeling and repeated calls, thus making it relatively expensive to compute. In the absence of collection equipment, public datasets and online searching are commonly used methods with the advantages of moderate data accuracy, low time consumption, and cost. The camera and video camera usually collect image data. High-priced equipment improves the resolution and accuracy of the image. Shooting thousands of photos takes months and requires the cooperation of equipment movement and lighting conditions. Later, UAVs are used to collect images in hard-to-reach places. Some researchers also take images by cell phone with moderate image accuracy, reducing costs somewhat.

## 4. Deep Learning Algorithms

**Figure 6:**
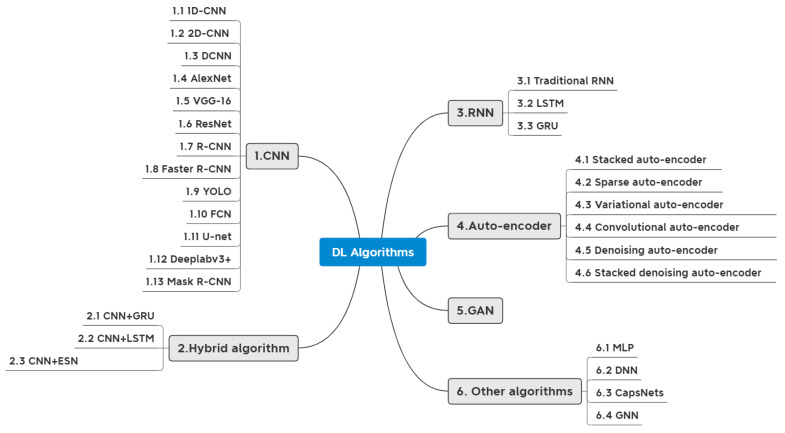
DL algorithm summary diagram.

Figure 6 summarizes the commonly used DL algorithms in SHM. CNN, RNN, auto-encoder, and GAN play different roles in DD, among which CNN is the most studied. There are five main steps in applying DL to SHM: (1) data type selection, (2) data acquisition, (3) data preprocessing, (4) feature extraction, and (5) damage detection. Further summary information about the algorithm types is shown in Table S2.

### 4.1. Convolutional Neural Network

#### 4.1.1. CNN

**Figure 7:**
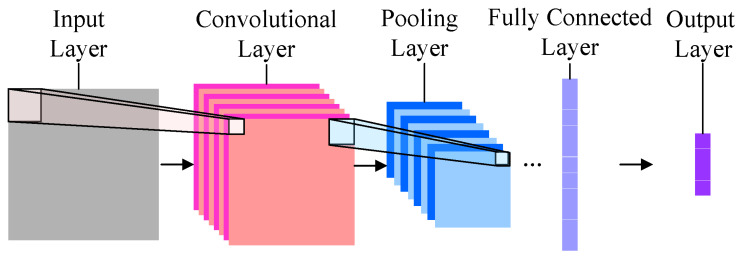
CNN architecture diagram [160].

The CNN is the most popular DL network variant, mainly applied to image processing. The architecture of CNN is shown in Figure 7, including the input, output, convolution, pooling, and fully connected layers. The input layer receives the image and passes it to the convolutional layer, which convolves the image using filters to achieve feature extraction. The pooling layer, also known as the sub-sampling layer, reduces the output size of the convolutional layer by calculating statistical features such as mean and maximum values. This layer can reduce the parameters and the amount of calculation. The fully connected layer uses a classifier to classify the image by combining all the previous layer’s information and then outputs the final result with the output layer. There have been various architectures in the development of CNN. Firstly, LeNet-5 [155] is a relatively early and simple architecture with seven layers for handwritten character image classification. AlexNet [156] successfully used rectified linear units as the activation function in CNNs, enabling deeper and broader CNN development. VGG-16 [157] adds depth by stacking convolutional layers and pooling layers, including thirteen convolutional layers and three fully connected layers. GoogleNet [158] consists of several inception modules, enabling dimensionality reduction and mitigating adverse effects such as gradient disappearance due to increasing depth. ResNet [159] builds deeper models and deals with the problem of accuracy reduction due to gradient disappearance through residual learning.

CNN’s processing of images can be roughly divided into three categories: image classification, object recognition, and semantic segmentation [100]. 2D-CNN is primarily used for image classification. For the input image, CNN uses a fixed-size sliding window to detect defects on each image block according to a certain stride [35]. Zhang et al. [161] applied CNN to classify bridge cracks into three categories: small, large, and serious, based on the crack images. In addition, when dealing with SHM problems based on vibration signals, GW, and AE signals, the method often adopted is transforming the signals into images in the time and frequency domains [37]. Wang et al. [120] adopted the Hilbert–Huang transform to convert the vibration signal into a time-frequency diagram as the input of a 2D-CNN for detecting seven damage modes of the IASC-ASCE benchmark structure. Chen et al. [21] processed the GW signal into a time-frequency map using a complex Gaussian wavelet transform and applied CNN to assess the length of fatigue cracks. Sikdar et al. [19] used Continuous Wavelet Transform (CWT) to process time-domain AE signals for AE source classification.

Region-based Convolutional Neural Networks (R-CNN) can solve the problem of object recognition. The traditional target detection method is the sliding window method. However, the size and stride of the sliding window are too large to reduce the detection accuracy and too small to increase the computational cost. Considering the above problems, Girshick et al. [162] proposed the R-CNN method. This method first determines the candidate region using a selective search and then extracts the defect features on the candidate region and identifies them with the bounding box to achieve object recognition. The successive introduction of Fast R-CNN [163] and Faster R-CNN [164] further improves computational efficiency. Pham et al. [165] used R-CNN to recognize bolts and backgrounds in synthetic images. Li et al. [166] realized the classification and location of coarse cracks in tunnel images through Faster R-CNN. Regarding the real-time detection problem, researchers subsequently proposed Single Shot MultiBox Detector (SSD) [167] and YOLO [168]. Hou et al. [134] employed YOLOv3-tiny and YOLOv3 to identify trucks, pickup trucks, and cars with high accuracy based on camera images of the effect of vehicle loads on bridges. Pan et al. [53] established Yolov3-tiny and Yolov3-tiny-KLT based on YOLOv3 architecture to realize vision-based structural vibration measurement.

For various defects, bounding box recognition cannot determine the shape of the defect well. In this case, pixel-level semantic segmentation can assign categories to each pixel in the image and separate the defect from the background to achieve more accurate detection and location. The models involved include FCN [169], U-net [170], FC-DenseNet [171], Deeplabv3+ [172], Mask R-CNN [173], etc. Wang et al. [41] used an FCN-based network to segment the steel beam crack splicing image to detect the size and location of the crack by the centroid coordinates, inclination, and other characteristic symbols. Zhang et al. [50] introduced a displacement monitoring method based on Mask R-CNN, which achieves the purpose of displacement detection by extracting the mask information to obtain the coordinates of the calibrated object. Qiu et al. [174] proposed that WRDeepLabV3+ combined with the class activation map could accurately identify leakage in subway tunnels, which could segment the leakage area more thoroughly.

The image processing of 2D-CNN involves a large amount of data, which has high complexity and requires special hardware equipment. Kiranyaz et al. [175] presented 1D-CNN, which had the advantages of low computational complexity, highly cost-effective, and real-time application in mobile devices compared to 2D-CNN. With the popularity of vibration signal-based SHM, vibration signals can be directly input into a 1D-CNN for damage identification, localization, and assessment. Zhang et al. [176] obtained the time-varying damage index from the Lamb wave and used the 1D-CNN to locate the damage in the plate. Sony et al. [1] divided the bridge damage into categories such as abutment settlement and tendon rupture using 1D-CNN based on vibration signals. Wu et al. [177] used 1D-CNN to process vibration signals from experiments and numerical simulations and then realized notch detection of steel beams.

#### 4.1.2. The Combined Application of DL Algorithms

For vibration-based SHM, the vibration signals collected by different sensors have strong temporal and spatial correlations. To effectively extract damage-sensitive features from vibration signals, it is necessary to adopt methods to process data from time and space dimensions. The problem of data temporal and spatial feature extraction can be solved using a combination of the CNN and RNN methods, such as CNN with GRU [64,109,123], CNN with LSTM [27,178,179,180,181,182], CNN with Auto-encoder and [183], CNN with echo state networks [113], etc.

Yang et al. [123] proposed a hierarchical CNN and GRU framework called HCG, which used a CNN to extract spatial and short-term temporal features and a GRU to learn long-term temporal dependencies. Through the evaluation of the IASC-ASCE and TCRF datasets, HCG is significantly superior to other existing damage detection methods in performance. On this basis, Yang et al. [109] proposed a framework for Parallel CNN and Bidirectional GRU, which avoided the loss of time-related features of data entering CNN first through dual-channel data processing. It is verified that this framework has better recognition performance than HCG on the same two datasets. Dang et al. [178] combined CNN with LSTM to process vibration signals, and they achieved high-accuracy bridge DD with reduced time and memory complexity. Parziale et al. [183] proposed a coupling method of a CNN and autoencoder to neutralize the influence of temperature change and improve the accuracy of damage detection on a limited dataset.

### 4.2. Recurrent Neural Network

**Figure 8:**
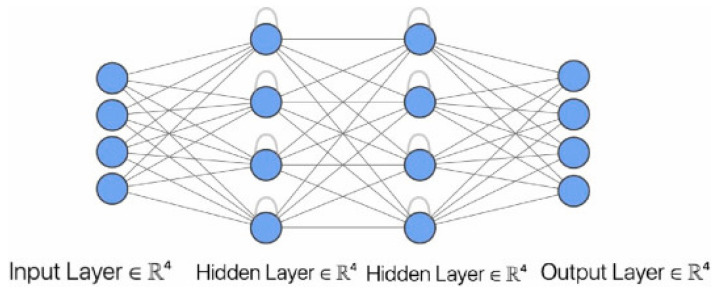
RNN architecture diagram [104].

RNN allows capturing the temporal dependencies between data and is suitable for sequential data processing, such as vibration data, GW data, etc. As shown in Figure 8, the RNN consists of input, output, and hidden layers. The key is the hidden layer containing multiple units, where the production of one unit depends not only on the input of this unit but also correlates with the output of the previous unit, allowing the information above to be applied to the current state. Back-Propagation Through Time (BPTT) is the standard training algorithm for RNNs, but the problem of gradient disappearance or gradient explosion occurs when using BPTT to solve long-time dependencies. The LSTM [184] is employed to solve this problem, using forgetting, input, and output gates as threshold mechanisms to manage information. Users can decide whether to add, delete, and pass information, processing longer sequences. GRU [185] is a variant based on the LSTM, which only contains an update gate and reset gate and has a faster training speed than the LSTM with fewer parameters.

Application of the RNN to SHM mainly uses time series data for damage identification, location, and evaluation. Relying on its advantage of capturing the time dependence of data, the RNN is also often adopted for missing data prediction. Based on the QUGS and Z24 bridge benchmark datasets, Sony et al. [61] first used LSTM to classify vibration signals into multiple categories. Then, the author presented the damage probability of all data through the heat map, and the area with high damage probability was the location where the damage occurred. Finally, the identification and location of multiple types of damage were realized using this method. Aiming at the random missing and continuous missing of dam data, Li et al. [186] exploited LSTM to capture the time dependence of the original sensor data to realize missing data interpolation. Deng et al. [187] used the GRU to establish a model of vehicle influence coefficient, temperature input, and deflection output, aiming to accurately predict the suspension bridge’s deflection.

### 4.3. Auto-Encoder

**Figure 9:**
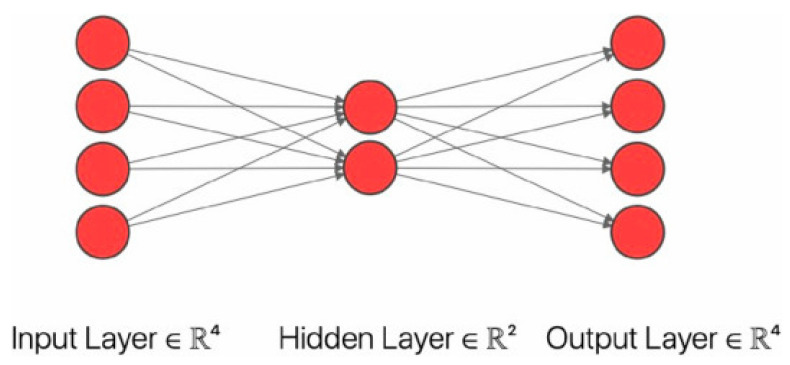
Auto-encoder architecture diagram [104].

Auto-encoder is an unsupervised learning model mainly applied to data denoising, dimensionality reduction, and feature extraction. As shown in Figure 9, the auto-encoder’s most straightforward architecture includes the input, output, and hidden layers. The input layer inputs data, the mapping of the hidden layer acts as an encoder to reduce the dimension and compress the data, and the mapping of the output layer acts as a decoder to reconstruct the data. The number of neurons in the input and output layers is the same [188], which aims to reconstruct the input data. According to different application requirements, auto-encoder includes many types, such as stacked auto-encoder, variational auto-encoder, denoising auto-encoder, sparse auto-encoder, convolutional auto-encoder, etc.

Auto-encoder performs data dimension reduction to eliminate unnecessary information such as data redundancy and noise in high-dimensional data and thus directly learn the necessary information. Pathirage et al. [189] introduced an autoencoder-based framework for structural damage identification, including dimensionality reduction and relational learning. The accuracy and efficiency of the framework were verified through numerical and experimental studies on the steel framework. Ebrahimkhanlou et al. [80] implemented AE source localization by stacking self-encoders with progressive compression of the input AE waveform. Coordinate localization or area localization was performed through regression or softmax layers, capable of localizing AE sources at arbitrary locations on the board. Lei et al. [190] studied a method for predicting the displacement of cable-stayed bridges using residual autoencoders. The method took temperature, wind speed, and vehicle load as input and displacement response as output, achieving more than 95% effectiveness.

### 4.4. Generative Adversarial Network

**Figure 10:**
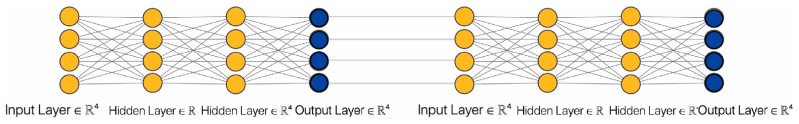
GAN architecture diagram [104].

The GAN is also an unsupervised learning method proposed by Goodfellow et al. [191] in 2014, mainly for image generation and data enhancement. The architecture of the GAN is shown in Figure 10. The GAN consists of two parts: a generator, which aims to synthesize data as similar as possible to the training data, and a discriminator, which aims to distinguish the original data from the synthesized data. The two oppose each other and improve each other. Traditionally, the generator is a multilayer perceptron, and the discriminator is a binary classifier. With the development of CNN feature extraction capability, CNNs can also be used as generators and discriminators, known as Deep Convolutional GANs (DCGAN) [192], improving training stability and effectiveness.

Chen et al. [76] employed the GAN to generate many virtual crack images similar to real images, which were applied to train CNN models for crack detection. Gao et al. [138] presented a balanced semi-supervised GAN that solved low data and imbalance class problems using balanced batch sampling during training. Rastin et al. [82] proposed a two-stage damage detection method. The DCGAN learned structural health data to obtain the probability of data from the structural integrity state, which was used to evaluate the severity of the damage. A conditional GAN classified the data into different accelerometer sources to achieve damage localization. Lei et al. [79] exploited the DCGAN to learn the features of the pre-loss data to reconstruct the lost data. Then, they distinguished the actual and reconstructed data using discriminators to improve the reconstruction accuracy.

### 4.5. Others

A Multi-Layer Perceptron (MLP) is an artificial neural network containing an input, hidden, and output layer. It is capable of performing classification and regression tasks. Due to the small number of hidden layers, it can only solve simple problems. Mariani et al. [10] adopted various methods of 1D-CNN, LSTM, and MLP for plate hole detection, among which the performance of the MLP method was slightly inferior. Deep Neural Networks (DNNs) are deep neural networks with more hidden layers than MLP, thus improving the performance of feature recognition. Kohiyama et al. [193] fully exploited the powerful DNN damage pattern classification capability and combined it with an SVM to achieve unlearned damage pattern classification.

Sabour et al. [194] proposed capsule neural networks, where each capsule consists of a set of neurons and is activated by a routing algorithm. It can solve the problem that the CNN cannot recognize the hierarchical structure in the image and has been applied to many fields to perform classification and regression tasks. Barraza et al. [195] employed CapsNets to localize and quantify the stiffness reduction damage in beams, showing that the method achieved better results than CNNs. Son et al. [196] proposed that a Graph Neural Network (GNN) can learn graph structure data, which overcame the limitation that a CNN can only learn grid structure data. Finally, using graph sensor data, the GNN was trained to successfully detect cable damage on cable-stayed bridges. Li et al. [197] proposed a transformer-based time series prediction framework, verified by bridge strain data, and was found to have a more minor error than LSTM.

### 4.6. Summary

**Figure 11:**
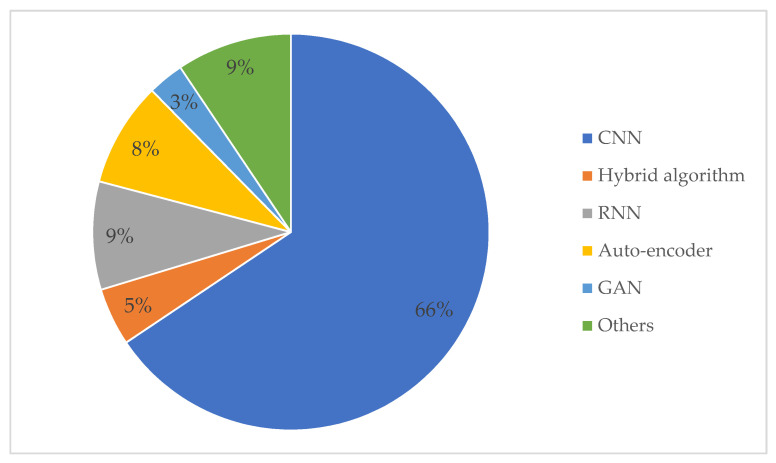
DL algorithm types percentage graph.

By summarizing the research literature at the algorithmic level in Supplementary Table S2, we clarify the percentage of DL algorithms in Figure 11. Figure 11 illustrates four common algorithms: CNN, RNN, auto-encoder, and GAN, where the CNN occupies a clear advantage regarding the number of studies. Based on the research and analysis of literature related to different algorithms, Table 3 is a detailed comparison of these four algorithms. Firstly, considering the data collected, the RNN cannot process images, while the other three algorithms can process images and time series data. Among them, time series data refers to data describing phenomena over time, such as vibration signals, AE signals, GW signals, etc. The CNN is excellent at processing images, and the RNN does well at handling time series data. The auto-encoder achieves data enhancement through data dimensionality reduction and denoising. The GAN offers the distinct benefit of expanding datasets by generating images. These algorithms have their applications, including object recognition, semantic segmentation, image classification, data enhancement, etc. In addition, the specific application functions, pros and cons of various algorithms are listed in Table 3 for reference.

The accuracy of DL models largely depends on the quantity and quality of data in the training and test sets. Sufficient and reliable data plays a vital role in DL application in SHM. Table 4 shows database examples of some of the literature, such as the size, nature, and ratio of the training set to the test set for vibration, GW, AE, and images when DL to SHM is applied. Large datasets have tens of thousands of samples regardless of the data type, while small datasets have only a few hundred samples. For example, Song et al. [51] used an FCN that inherited all the connection weights of the VGG-16 trained on PASCAL VOC2012 and, therefore, required less data fine-tuning of the pre-trained FCN. The function of the test set is to evaluate the performance of the trained DL model. Generally, the training set’s ratio to the test set is 8:2 [27,36,182,198] or 7:3 [15,58,187]. Also, validation sets were added to adjust the DL model parameters better. The typical ratios of the training set, verification set, and test set are 6:2:2 [17,110], 7:2:1 [22,44], 8:1:1 [128,199] and so on.

The performance of different DL algorithms varies, especially when using the same dataset. Many indexes can measure the performance of the DL algorithm, such as accuracy, precision, recall rate, Mean Average Precision (MAP), etc. These indicators can be used to compare and quantify the detection results of different DL algorithms. Table 5 shows examples of literature comparing the performance of multiple DL algorithms on the exact damage detection task. By comparison, the optimal model can be found on the one hand, and on the other hand, whether the newly proposed framework can achieve satisfactory performance can be verified. For example, Arafin et al. [132] compared the performance of multiple CNN networks, namely VGG-19, ResNet-50, InceptionV3, Xception, and MobileNetV2. The accuracy, precision, and recall rates of the InceptionV3 model were higher than those of other models. Wang et al. [44] found that among YOLOX, Faster R-CNN, Deconvolutional Single Shot Detector, and YOLOv5, the MAP of YOLOX in crack detection was 88.5%, showing the best performance. Liao et al. [122] proposed a channel-spatial-temporal attention-based network to detect vibration-based damage. The framework outperformed the latest DL methods, such as CNN, LSTM, HCG, and FCN, through implementation verification on the TCRF and benchmark datasets.

## 5. Application Objects and Functions

**Figure 12:**
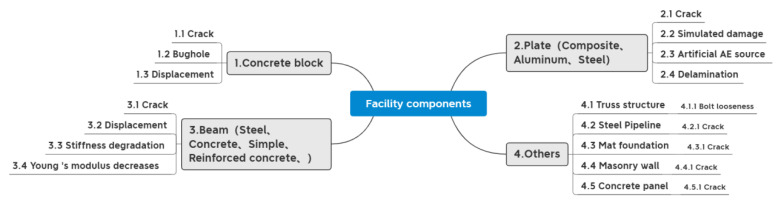
Application summary diagram of facility components.

**Figure 13:**
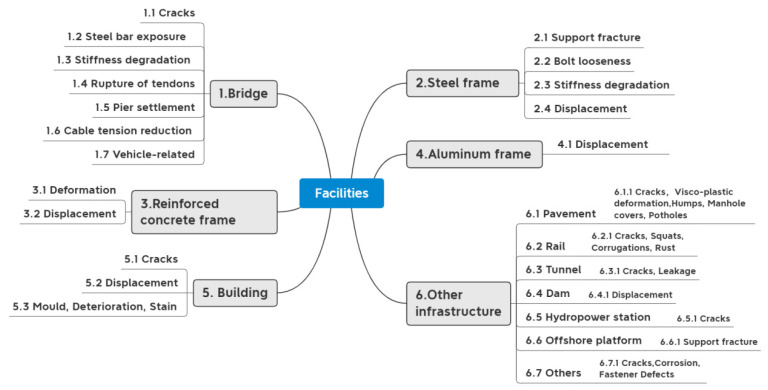
Application summary diagram of facilities.

**Figure 14:**
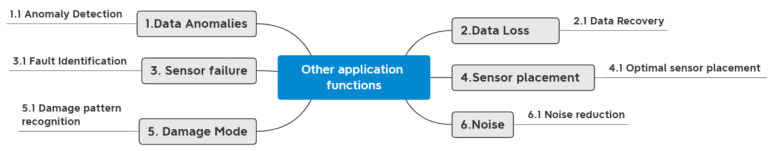
Other application functions summary diagram.

The research object of this paper is not only facilities but also facilities components. Under each category, different application functions and DD levels are divided. The application functions of the different facility components and facilities are summarized in Figure 12 and Figure 13. Figure 14 illustrates additional application functions such as data anomalies, sensor placement, noise, etc. The detailed contents are shown in Tables S3–S5. In this paper, DD in SHM is classified into three stages: identification, localization, and assessment. DL can solve the binary classification problem of whether the damage exists or not, the multi-classification problem of different damage severity, and determine the location of the damage.

### 5.1. Facility Components

#### 5.1.1. Concrete Block

Concrete blocks are frequently used for research in the laboratory, which facilitates the control of experimental conditions and the acquisition of experimental data to validate the proposed algorithms. Damages studied include concrete cracks, bugholes, alkali-silica reactions, and displacement monitoring, which is difficult to achieve in large structures. For the crack, Jang et al. [145] verified that a trained Deep Convolutional Neural Network (DCNN) could achieve macroscopic and microscopic crack recognition and visualization using crack images of concrete blocks in the laboratory. Siracusano et al. [62] converted the acoustic signals into electrical signals by installing AE sensors on the test blocks. Then, they input the signal to the LSTM to identify the presence of cracks and classify them into three types: tensile, shear, and mixed.

Holes in concrete surfaces can firstly affect the aesthetics and, more importantly, cause the debonding of fiber-reinforced polymers [206]. The salt in the holes will accelerate the degradation of the structure [207], so detecting the holes as soon as possible can improve the structure’s durability. Wei et al. [208] captured the surface hole image of standard concrete specimens to form a dataset and trained a DCNN to automatically identify holes, with an accuracy rate of nearly 90%.

The displacement of the structure is one of the indexes to evaluate the system’s safety. Once exceeding the design value, it may cause the collapse of the building. Sensor or vision-based techniques are often used for displacement monitoring [50]. Zhang et al. [209] took images of concrete blocks with black markers by cell phone. Then, an FCN was employed to identify the marked area and obtain the center coordinates to achieve displacement monitoring at a distance of 10 m.

#### 5.1.2. Composite Plate and Metal Plate

Composite materials, such as Carbon Fiber-Reinforced Polymer (CFRP), have high strength, low density, corrosion resistance, and fatigue resistance [210]. However, the composite structure is prone to delamination, fiber breakage, and other damages under the external impact load, resulting in decreased compressive strength. Thus, it is necessary to identify the location and size of the damage as early as possible. Because UGW can propagate well in the composite plate while having high sensitivity to defects, the UGW signal is widely exploited to detect defects automatically. Ijjeh et al. [210] transformed the full-wave field data of the composite plate into a root mean square image and segmented pixel by pixel using the FCN without feature extraction. Finally, the delamination was identified and marked as red. Cristiani et al. [211] converted the distributed fiber optic sensors to collect strain data from 1D to 2D and achieved delamination prediction of the CFRP specimens via CNN.

Due to the working environment and load conditions, metal plate structures such as aluminum and steel are prone to damage, including fatigue cracks, gaps, holes, corrosion, failure, etc. In laboratory studies of metal plates, researchers generally use metal nuts, mass blocks, and aluminum strips attached to the plate surface to simulate cracks, gaps, and other damages. The SHM method extracts damage-sensitive features from AE and ultrasonic signals using DL methods such as CNN for DD. Hesser et al. [20] formed three different AE sources using the Hsu–Nielsen pencil core fracture source, 3 mm, and 5 mm ball impact. The collected AE signals were input into the ANN, 1D-CNN, and 2D-CNN for evaluating the ability of different methods to identify different types of AE sources. Zhou et al. [212] pinned cylinders of different masses on stainless steel plates, collected UGW signals using PZT sensors and inferred multiple damages and their respective positions by the WaveNet model. Miorelli et al. [213] drilled a hole with an increased radius on the aluminum plate. They collected Lamb wave data through numerical simulations and experiments to train and verify CNN’s ability to locate and evaluate holes.

#### 5.1.3. Steel and Concrete Beams

The beam is also a common facility component, including steel beams, concrete beams, reinforced concrete beams, etc. These beam structures are made into simple, cantilever, continuous, and other forms to study damages and model algorithms. Damage such as cracks, displacement, and stiffness reduction can occur in steel and concrete beams. Compared with the excellent results of the UGW in sensing damage in plate-like structures, the vibration-based damage identification method is more suitable for beam-like structures. Researchers employ different DL algorithms to identify the relationship between vibration characteristics and structural damage for damage identification, localization, and assessment. Seventekidis et al. [214] collected acceleration signals to verify the ability of a 1D-CNN trained using simulated data to identify additional damages. Displacement can be easily monitored using computer vision methods to track critical points of the structure through images and video. Luan et al. [215] proposed an optical flow method that extracted full-field displacement from aluminum cantilever beam video through two CNN architectures with high accuracy.

Concrete and reinforced concrete beams are susceptible to crack damage, which can be detected by DL image processing. Ye et al. [216] trained an FCN for crack recognition and verified the better performance of the FCN over edge detection using crack images of indoor concrete beams. Kong et al. [217] employed CNN to identify and segment cracks from steel-concrete-steel sandwich composite beam images. They also proposed coarse matching and precise matching algorithms for labeling cracks to identify old crack extensions and new crack generation. Nguyen et al. [218] developed a 1D-CNN model to extract and learn the optimal features from the original electromechanical impedance signals to assess the severity of prestress loss in reinforced concrete beams.

#### 5.1.4. Others

In addition to the block, plate, and beam structures, the facility components used in laboratory research have other forms for targeted research. These research objects face different damages and need to explore corresponding solutions.

Seventekidis et al. [219] constructed a FEM to generate vibration data and input it into a CNN to achieve the CFRP-hinged truss bolt loosening. They identified the damage using hierarchical binary classification. For the cracking of raft foundations, Han et al. [220] proposed a method to identify cracks using AE signals emitted during cracking and a trained 2D-CNN to identify fissures. Hallee et al. [221] studied the masonry structure cracking problem using images with mortar joints and trained CNNs with three different damage-recognition architectures. Nguyen et al. [218] designed 16 damage states for steel pipes and used a CNN to achieve high-precision detection of different welding types, positions, and severity.

### 5.2. Facilities

#### 5.2.1. Bridges

As one of the critical infrastructures, bridges play an increasingly important role in transportation with an increase in the number. During the long-term use of bridges, negative impacts from traffic loads, wind loads, material deterioration, and sudden disasters can cause various damages such as cracks, exposed reinforcement, broken ties, settlements, etc. These damages will reduce bridges’ bearing capacity and service life and even cause incalculable losses [222]. SHM is crucial and indispensable for bridges because it can detect damage as early as possible to avoid loss. At present, several bridges in China have been installed with SHM systems, such as the Sutong Bridge in China [223,224], the Yonghe Bridge in China [124], and the Canton Tower [225]. The SHM system uses sensors to collect large amounts of data for real-time monitoring of bridge conditions.

Cracks are one of the most basic damages, making them a research hot spot. The commonly used methods in research literature include vibration-based methods and image-based methods. The vibration-based method considers the change in vibration characteristics caused by damage, such as cracks, so that the evolution and position can be detected by feature extraction. The image-based method detects cracks in the image through an image processing algorithm. DL algorithm promotes the development of these two methods. The vibration acceleration signal is a time series signal. Yang et al. [109] considered damage identification based on the acceleration signal as a multivariate time series classification problem. They used the CNN and GRU for parallel feature extraction to obtain temporal and spatial correlations, which were validated using the IASC-ASCE and TCRF datasets with an accuracy of 94.92%. Considering the correctness of the model detection effect, Sajedi et al. [144] employed a deep Bayesian network for image-based impairment recognition while outputting the prediction results with the uncertainty of the results. They found that uncertainty and classification errors were closely related and intervened when uncertainty was high.

In different positions of the bridge, there will be a variety of damage in addition to cracks. Firstly, as the number of vehicles increases, traffic congestion, temperature, wind, and other environmental effects, the bridge will suffer from defects such as steel bar exposure, tendon rupture, pier settlement, stiffness reduction, cable tension reduction, material deterioration, etc. Both concrete delamination and rebar exposure occur on the surface of bridges so that a DL-based image segmentation method can be applied for DD. Rubio et al. [49] collected bridge deck images from the infrastructure inspection records of 26 cities in Niigata Prefecture, Japan, and implemented semantic segmentation using FCN to accurately identify delamination and rebar exposure. Sony et al. [61] proposed a windowed LSTM method to identify and locate various types of damage, including tendon rupture and pier settlement, through the vibration response of the Z24 bridge dataset. Sarwar et al. [226] developed a numerical model of a vehicle-bridge interaction system in which damage was modeled as varying degrees of stiffness reduction. The responses generated by the model were utilized for training a deep auto-encoder to implement bridge damage assessment. Dang et al. [125] used the vibration signals of a cable-stayed bridge to detect the location and level of tension reduction. They compared four different DL methods, MLP, LSTM, 1DCNN, and 2DCNN, for damage identification, localization, and evaluation, with the 2DCNN providing the best results. Toan et al. [227] proposed a CNN to evaluate energy dissipation to monitor the material degradation of the Saigon Bridge in Vietnam.

With the rapid growth of traffic volume and the frequent occurrence of vehicle overload, the influence of load brings a significant threat to the bridge’s safety. Monitoring vehicle loads becomes a non-negligible option for bridges. A common technique nowadays is the Bridge Weigh-In-Motion (BWIM) system proposed by Moses [228], which exploits the bridge strain response to estimate the vehicle weight. Ge et al. [229] presented the bridge traffic load monitoring system, which integrated the information of the WIM and camera. YOLOv3 can determine the vehicle’s position by identifying the centroid and size of the vehicle. Then, they combined the weight and position of the vehicle in real time to clarify the load distribution of the bridge. However, the BWIM system requires expensive hardware facilities [135], including sensors and data acquisition systems, which cannot be widely used due to cost considerations. Thus, there have been researchers investigating other methods to detect vehicle weight. Zhou et al. [135] classified vehicles into nine types and determined the weight information corresponding to each type. Then, they trained AlexNet and VGG-16 models for vehicle classification and Faster R-CNN for vehicle identification and location in surveillance videos to obtain the weight of each vehicle.

#### 5.2.2. Frame Structures

The frame structure includes a steel frame, reinforced concrete frame, and aluminum frame, and SHM is mostly used in steel frames. The main reason is that the International Association for Structural Control (IASC) and the American Society of Civil Engineers (ASCE) provide SHM benchmark data as a unified platform to verify and evaluate the performance of various SHM methods. This benchmark data was taken from a four-story steel frame for which damage was simulated by removing braces and loosening bolts. In addition, Johnson et al. [230] developed different FEMs to simulate the benchmark structure and generated response data for various conditions by MATLAB. Abdeljaber et al. [231] introduced a new method based on a 1D-CNN. The technique used two sets of data from the IASC-ASCE benchmark data, case 1 (undamaged) and case 9 (severely damaged), to train 12 CNN classifiers. It estimated the overall damage to the structure by calculating the probability of damage values.

In addition to the benchmark structure, other types of steel frames aim at damage, such as bolt loosening and stiffness reduction. Firstly, the problem of bolt loosening is mostly a two-classification problem, i.e., loosening and tightening, which belongs to damage identification. If the goal of DD is to detect the degree of loosening, it is classified as damage assessment. Zhao et al. [232] trained the lightweight CNN model MobileNets to identify the angular coordinates of bolts from images for calculating the bolt loosening angle. The algorithm can be embedded in smartphones. Stiffness is a vital damage index label for structures. The degree of stiffness reduction is correlated with the severity of the structural damage. The nonlinear relationship between the structural modal information (natural frequency and vibration mode) and structural damage can be studied using stiffness. Damage assessment is achieved by inputting modal information and outputting stiffness cases through DL methods. Wang et al. [117] suggested a deep residual network to map the vibration response obtained from the steel structure numerical model to the stiffness reduction, which improved the evaluation performance. Oh et al. [233] trained the CNN with the health data of the steel frame and compared the differences between the health data and the output data to identify the damage and its location.

The damage to the reinforced concrete frame includes deformation and displacement. Sajedi et al. [234] developed a fully convolutional encoder-decoder neural network to classify 16 damage mechanisms in the 10-layer reinforced concrete frame numerical model. Aluminum frames target issues, including displacement and reduced stiffness. Morales-Valdez et al. [235] presented the frequency-domain CNN and principal component analysis methods. Firstly, they collected the data when the bolts of the aluminum frame were loose and estimated the lag displacement through the CNN in the frequency domain. Then, input acceleration and lag displacement to the CNN classifier can improve the accuracy of the damage location.

#### 5.2.3. Other Buildings and Infrastructure

There will be cracks, potholes, deformation, and various surface defects on the road surface, which significantly affect the safety and comfort of the vehicle. Frequently used methods for pavement fault detection include laser-based methods, where laser data are used to obtain road scan data for crack detection [69,236]. Image-based methods combine DL for image classification and object detection [144,237]. Vibration-based methods detect pavement defects by detecting time series anomalies [238]. Roberts et al. [237] manually labeled images captured by cell phones into four types: cracks, deformations, surface defects, and others, and implemented damage classification by the Faster R-CNN model and SSD model.

The location of rail failures includes rails and fasteners. Rail can fail with cracks, squats, corrugations, rust, and other faults. Fasteners can suffer from loose bolts and deteriorating under-rail pads. These defects can lead to train derailments and injuries. Research on railroad SHM has focused on image and AE-based methods. Iyer et al. [239] proposed a rail fault detection system. The system used robots to detect cracks, squats, corrugations, and rust by ultrasound, followed by images taken by a camera. Then CNN identified the defects, and GPS located the defects. The AE method can monitor the growth of rail cracks, but it is susceptible to noise interference and has a poor detection effect on small cracks. Chen et al. [146] trained the lower layer of the CNN on the online audio database, and the other layers were trained on the rail AE data so that the model could be fully learned to achieve accurate monitoring of cracks. Chen et al. [240] input the axle box acceleration, the track irregularity, and the vehicle speed into the FCN to evaluate the damage degree of rail fasteners.

Tunnels can be affected by cracks, leaks, spalling, and other damage. Traditional visual inspection methods have the disadvantages of inaccurate results, dangerous operating environments, suspension of operations, and low cost effectiveness. Then, cameras, radar, lasers, and other methods are mostly adopted to capture images. Ren et al. [241] introduced a pixel-level segmentation network, CrackSegNet, which can precisely segment the shape and location of the cracks. However, the dark environment of the tunnel and the presence of pipes, cables, and stains can influence image quality and detection accuracy. Attard et al. [242] introduced a shading algorithm to correct the images for light and segmented the reflective areas of pipes for shielding using U-net, thus reducing recognition errors.

The dam is a vital infrastructure that can play the role of flood control, water supply, and power generation. It often faces extreme weather and loads during operation, resulting in deformation and dam failure. Many dams are equipped with SHM systems, such as the Ertan and Jinping dams in Sichuan Province [186], to monitor the operating conditions of the dams in real-time. The DL method is introduced to predict dam displacement as an index to measure dam deformation. Li et al. [65] decomposed the time series of dam displacement into seasonal, trend, and residual components, adopted extremely randomized trees and stacked LSTM to predict the displacement of each component, and finally obtained the total displacement of the dam. In addition, they also studied the CNN to predict displacement through environmental monitoring data [64] and the method of stacking the LSTM to predict various missing data of dams through adjacent sensors [186].

Offshore platforms undertake the task of offshore operations in complex marine environments, so damage will inevitably occur. Brace damage will occur on conduit-frame offshore platforms, and vibration-based damage detection methods can achieve excellent detection results. Bao et al. [243] developed a 3D catenary offshore platform model to simulate single and multiple location support damage, followed by a 1D-CNN to detect damage location and severity based on strain data. Puruncajas et al. [244] simulated cracks and bolt loosening on jacket-type foundations of offshore wind turbines laboratory scaled-down devices. Furthermore, they converted the collected acceleration signals into images for the CNN to implement damage identification.

### 5.3. Other Application Functions

Sensor failures, transmission failures, harsh environments, and other effects can cause abnormal data, directly affecting DD accuracy. Later, a new research topic for SHM emerged: data science, including data cleaning, data compression, data recovery, data fusion, and data interpretation [59]. Among them, data cleaning is the process of anomaly detection and removal, which is the focus of the current research. Son et al. [67] developed an LSTM-based encoder-decoder architecture to process time series data and calculate anomaly scores. Then, temporary errors were identified and removed according to the abnormal scores. Considering that time series data are insufficient to distinguish specific anomalous patterns, Tang et al. [223] trained a CNN by converting acceleration data of large-span cable-stayed bridges into time-frequency images and identified six anomalies, such as loss, trend, and drift. The conversion of 1D data to 2D images increases the processing time, so Jian et al. [59] converted 1D acceleration data to the inverse envelope of the relative frequency distribution histogram, which was input to a 1D-CNN to detect five anomalies. For the four fault types of missing, spiky, random, and drift, Jana et al. [245] used a CNN to identify the existence and type of faults and then reconstructed the faults using the convolutional autoencoder.

Data loss is the most frequent type of data anomaly, accounting for many research articles. Fan et al. [246] worked with a CNN to find the nonlinear relationship between the acceleration signal with transmission loss and the complete signal, thereby outputting the recovered original signal. Li et al. [247] decomposed the original data into multiple intrinsic mode functions by empirical mode decomposition. They successfully employed LSTM to predict each IMF’s missing values and aggregated them to recover the lost value. For the unsupervised approach, Lei et al. [79] trained deep convolutional GAN models using data obtained from intact structures and performed an excellent reconstruction of the missing signals in numerically simulated acceleration data and experimentally measured strain data. SHM relies on a large number of sensors to obtain structural information. Li et al. [68] proposed optimal sensor placement to enable global information collection using limited sensors and reduced costs. Sajedi et al. [248] proposed that Deep Generative Bayesian Optimization (DGBO) could reduce the number of sensors used for damage localization and evaluation by 52% and 43%, respectively. Huang et al. [249] considered the effect of ambient white noise in the field when predicting the mass of a conduit frame. A denoising self-encoder was used to reconstruct the input variables to achieve the denoising of vibration signals.

### 5.4. Summary

**Figure 15:**
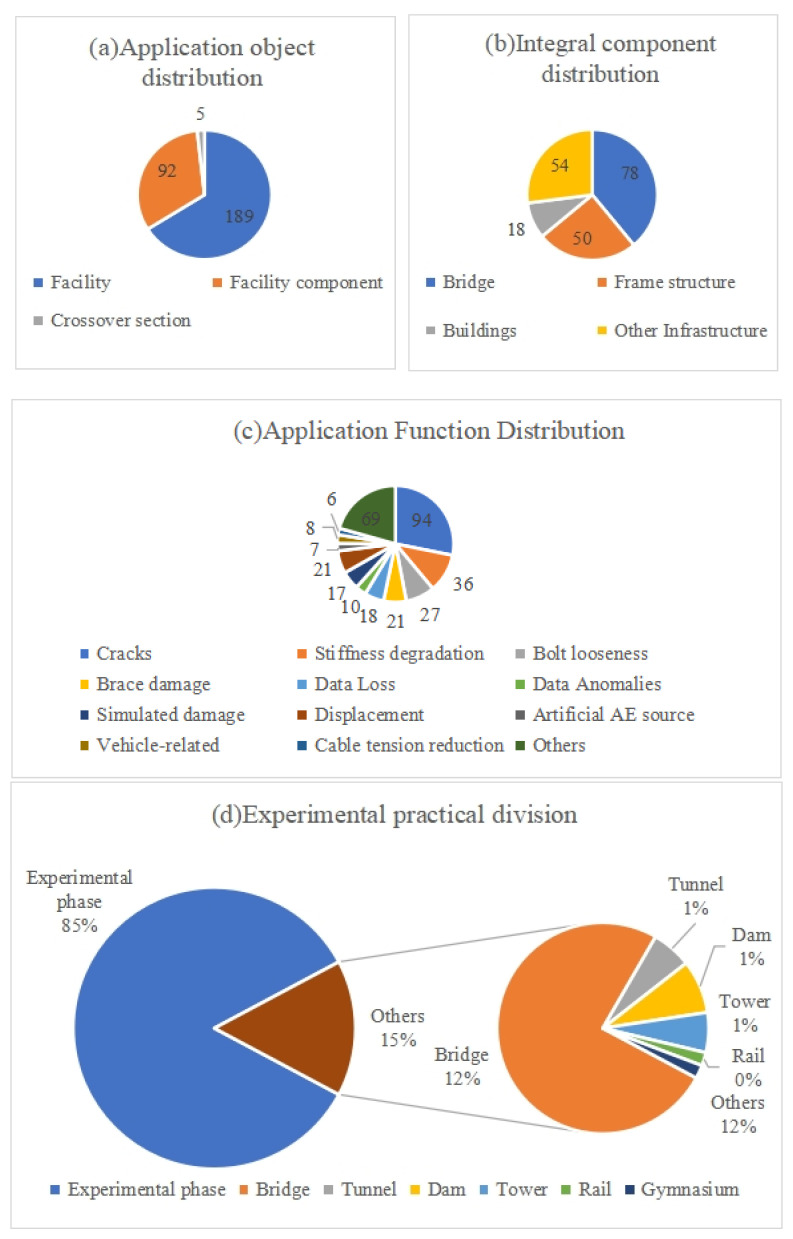
Application summary diagram.

Supplementary Tables S3–S5 summarize the research literature at the application level, categorizing it according to different facility types, application functions, and application results, and then obtaining the percentage of facility types. Given this, the literature was further analyzed to form Figure 15, which reflects the percentage of all research literature in terms of application objects, application functions, and the percentage of experimental and practical stages. Firstly, facilities dominate the application objects. Some papers have conducted comparative analysis by simultaneously studying facility components and facilities. Next, facilities are categorized into four parts: bridges, frame buildings, buildings, and other infrastructure. Much attention has been paid to frames and bridges, and some researchers have conducted individual studies of infrastructure such as roads, railroads, tunnels, and dams. The previous section examined the various application functions of the different application objects, while the diagram below highlights the application functions of all articles. Researchers are concerned with cracks in various structures and assess structural damage through stiffness reduction. Secondly, many articles have analyzed two types of damage in benchmark steel structures, i.e., loose bolts and brace damage. Researchers have begun to focus on data loss and anomalies since abnormal data is connected to the accuracy of subsequent DD. Both the number of connected studies and the solutions are gradually growing. In addition, some targeted studies on simulated damage, displacement, artificial AE sources, vehicles, and cables are also involved.

The application phase of the research can be categorized into the experimental phase and the practical application phase. Specifically, constructing models, collecting data, and detecting damage in the laboratory is called the experimental phase. Evaluating the damaged condition of the structure using actual structural data is called the practical application stage. It can be seen from Figure 15 that only a small part of the research literature has reached the practical application stage, and bridge research is the focus of this part. The Bridge Structure Health Monitoring (BSHM) system, such as the Tianjin Yonghe Bridge and Vietnam Saigon Bridge, was established. There are also government-retained bridge maintenance data, such as Japan’s Niigata government bridge inspection records and South Korea’s bridge inspection history database. Other practical structures include the dam, tunnel, railroad, and Canton Tower, the data of which can verify the effectiveness of the proposed method.

## 6. Discussion

### 6.1. Challenges

DL has injected new advantages and opportunities for SHM. However, a study of the searched literature reveals some challenges for DL-based SHM. The approach can be genuinely applied to infrastructure monitoring and maintenance if these challenges are effectively addressed in the future.

#### 6.1.1. Data Issues

(1) Data shortage

Although DL algorithms have higher performance than ML, a large amount of labeled data is needed to ensure model performance when the DL algorithm is used for image classification [131], location [44], and segmentation [201], resulting in data shortage. Gao et al. [138] mentioned that the computer vision baseline dataset (ImageNet with 14 million images) has more images than the SHM baseline dataset (PEER Hub ImageNet with 36,413 images). The main reasons are the limited number and type of sensors deployed on large structures and the difficulty of installing or taking images in hard-to-reach locations. Secondly, the data obtained from the actual structures often have a large proportion of “normal” data and only a small proportion of “damaged” data. There is a lack of “damage” data and class imbalance problems. These problems make the DL damage model inadequately trained, with reduced detection accuracy and even overfitting [138]. Abdeljaber et al. [231] mentioned that collecting data on all possible damaged locations of large civil structures is difficult, resulting in the lack of “damage” data. Finally, most datasets studied so far focus on one data type. In contrast, diverse data can detect different damage types and even react to the structural state from different perspectives. To sum up, lack of data is the common and primary problem of DL applications in SHM. The corresponding solutions are FE and unsupervised algorithms mentioned in the trend section.

(2) Data Loss

In the research literature, 22 pieces mentioned the data loss problem, which is detailed in Supplementary Table S5. According to literature analysis, the reasons for data loss are complex, including sensor failures, reduced accuracy, power outages, and interruption of wireless sensor transmission networks caused by changes in the external environment and long-term use of sensors. For example, Fan et al. [246] mentioned the signal loss of the wireless sensor network installed on the bridge related to radio interaction and signal attenuation during transmission. Loss of critical information will directly affect the accuracy of damage identification and judgment of the structure’s condition, which may be detrimental to the long-term monitoring of the infrastructure. Timely remedial measures must be considered to restore and reconstruct the structural response at the lost locations as soon as possible. Liu et al. [250] mentioned the method of recovery using original sensory data and the interpolation method using estimates to replace missing values. Researchers have also used DL algorithms for data recovery [71,128], but more effort is needed to address data loss at the source.

(3) Data Quality

Through literature analysis, data quality is summarized as data anomaly, noise, and image quality. As shown in Table S5 in the Supplementary File, 13 articles dealt with data anomalies, and two articles dealt with noise reduction. According to 13 relevant papers, data anomaly refers to outliers, trend items, and random drift in the case of sensor failure, system anomaly, or environmental factors [223]. Liu et al. [69] considered the distinction between sensor failure and structural damage, mainly by detecting the difference between the measured and reconstructed values of the sensor. Some studies use laboratory-acquired data without considering the effect of noise, while data collected in real-world environments are bound to contain noise. Suitable methods are needed to separate and handle noise without affecting the performance of the detection algorithm. Fan et al. [251] mentioned that noise in vibration signals includes environmental, measurement, and instrument noise. Moreover, DL can extract noise-insensitive features to achieve signal denoising. In addition, uneven illumination, equipment movement, and weather conditions are the main reasons for the decline in image quality. The effect of vision-based SHM is closely related to image quality. Improving image quality can avoid false negatives and positives as much as possible. Attard et al. [242] fully considered the influence of uneven illumination, stains, pipes, cables, and other factors in the tunnel environment and then adopted image fusion and DL methods to realize the damage detection of tunnel lining cracks.

(4) Data Storage

The implementation of SHM systems relies on a large number of sensors. With the development of long-term monitoring, data increases and accumulates dramatically, becoming a ‘big data’ problem. For example, the data volume of the Sutong Bridge in China reaches 2.5 TB per year [223]. Thus, it is necessary to increase the storage space and select the appropriate data compression method and powerful data processing technology to improve the efficiency and ability of data storage and processing. Ni et al. [129] proposed that appropriate data compression methods should be adopted to store data effectively. Particular attention should be paid to the compression and reconstruction of abnormal data, which requires using CNN and autoencoder methods. In addition, the data can be collected randomly through the compressed sampling method to reduce the amount of data. Bao et al. [102] mentioned that most vibration signals of infrastructure are sparse, so data can be collected by compressed sampling to reduce the sample size. Then, a sparse reconstruction algorithm was used to reconstruct the original signal.

#### 6.1.2. DL Performance

(1) Overfitting

A direct consequence of overfitting in DL is good performance on the training set but poor performance on the validation and test sets. One of the reasons for this phenomenon is data problems, including less corrupted data and noise effects. Zhou et al. [252] pointed out that overfitting would occur when the number of training datasets was less than the number of model training parameters. Secondly, when there is more noise in the signal, the DL model may mistakenly learn the features of noise, resulting in overfitting. Ibrahim et al. [253] proposed a method to use a high-pass filter to eliminate noise effects and improve the generalization ability of the CNN model. Finally, the model is too complex, and the training time is too long, which can easily lead to overfitting. To solve the overfitting problem, the dataset should be expanded using data enhancement (normalization, rotation, etc.), GAN-generated data, and data noise should be reduced. Regarding the model, regularization, weight sharing, dropout, and batch normalization are used to reduce model complexity. The training process can set baseline standards and draw on integrated learning methods.

(2) Model Architecture Selection

There are two critical steps in the execution of applying DL to SHM: the selection of features and the selection of classifiers. After determining the feature type, realizing the classification through the appropriate classifier is a continuous combination and testing process until the best choice is found. Moreover, it is necessary to consider various parameters that affect the model’s performance, such as the number of network layers, iterations, etc. At present, the determination of the optimal model architecture is mainly achieved through repeated testing. Now, researchers have also begun to study optimization techniques to determine the optimal architecture of the model. Oh et al. [254] used multi-objective optimization techniques to automatically search the values of kernel size, down sampling size, and layer depth to derive an accurate and efficient CNN architecture. Alazzawi et al. [255] proposed improving the network’s performance through Bayesian optimization technology.

(3) Credibility of results

Although the DL algorithm has excellent DD ability, it depends on the quantity and quality of data. Considering the small dataset and class imbalance problem mentioned above, the possibility of errors in the output of DL algorithms cannot be ignored. When applying DL in SHM to actual bridges and other infrastructures, detection errors can lead to serious consequences and high costs when they occur. Therefore, it is necessary to apply a method to determine the uncertainty of the model for application reference. Sajedi et al. [126] proposed a double Bayesian inference method to achieve robust damage diagnosis on degree-unbalanced datasets.

(4) Black box characteristics

Similarly, considering the credibility issue, the interpretability of the DL algorithm is now required to explain how the final test results can be obtained. This type of model is also called a black box. Gao et al. [256] mentioned that failure to understand the discriminant principle of the DCNN model makes its working principle a black box, which may lead to wrong decisions and serious consequences. Therefore, DL algorithms that can be understood, explained, and trusted must be studied. In this way, we can better understand and use the DL model by explaining the model’s decision-making process, thus promoting its rapid development.

#### 6.1.3. Practical Application Obstacles

(1) Application cost

Cost is a realistic problem for SHM applications that must be considered from several aspects. Firstly, collecting data is expensive for SHM systems, which deploy many wired and wireless sensors and are equipped with cameras or drones. Secondly, DL relies on high-performance graphics processing units, tensor processing units, and computer-unified device architectures to process data [141], which are costly to equip with hardware devices. Therefore, it is necessary to study low-cost SHM detection methods. For example, Roberts et al. [237] proposed a low-cost road detection method that uses smartphones to capture images instead of expensive laser imaging equipment, providing road management tools for road authorities.

(2) Application number

As shown in Figure 15, the literature on the practical application of SHM only accounts for 15% of the research literature, mainly concentrating on bridges. For example, Rastin et al. [121] used acceleration data collected by the SHM system of Yonghe Bridge in Tianjin to detect pier damage. The reasons for the low number of applications are twofold. First, there are many issues to consider with SHM, including the physical installation difficulty, building importance, data management and analysis capabilities, construction cost, operation period, etc. Second, as research on DL algorithms increases the network’s depth and improves the accuracy of the results, it is necessary to increase the hardware and computing power. Correspondingly, it also increases the difficulty of porting to smart devices [257], leading to application limitations.

(3) Application unity

The study of the retrieved literature shows that most articles are based on specific structures and tasks. The DL algorithm is also designed to detect particular damage. However, whether the method of bridge crack identification can be applied to dam crack identification or even bridge deformation identification is a concern. There is a need for a generalized DL approach suitable for various structures and tasks. Zhang et al. [258] successfully applied the damage level detection model to damage location detection through the multi-task network training strategy to achieve multi-task monitoring. In addition, the methods proposed by different researchers are independent. Comparative studies are difficult because they use self-built datasets, custom tags, etc., making finding problems hard. Publicly available benchmark datasets can solve this problem. Ye et al. [222] constructed an image dataset called the bridge crack library, which collected 11,000 high-pixel labeled images of 50 Bridges in 2 years and was verified using a DNN for crack detection.

### 6.2. Trends

**Figure 16:**
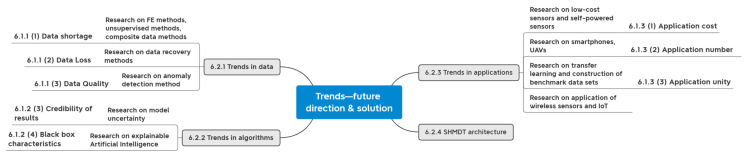
Trends (future direction and solution) summary diagram.

The trend part is the future research direction and the solution to the current challenges. As shown in Figure 16, the trends section is divided into four aspects: data, algorithms, applications, and the SHMDT architecture. The first three aspects are the specific answers to the questions in the challenge section of the data, algorithms, and applications. Each of these points is also a possible future research direction for the application of DL to SHM.

#### 6.2.1. Trends in Data

(1) Research on FE methods, unsupervised methods, and composite data to cope with the lack of data

DL algorithms’ rapid development is accompanied by a high demand for structurally damaged data. Destroying the structure to study the algorithm’s performance under different damage modes is impossible. The numerical simulation of FEM can generate data for almost all the required damage scenarios for the DL algorithm. Even the nonlinear relationship between different responses can be analyzed by FE to reconstruct the response of the lost data. Combining FE and DL to form a data model co-driven approach has become a key to future research. However, FEM involves the problem of model updating, which requires minimizing the difference between the model and the structure to update the model parameters [225]. Thus, accurate data close to the actual situation can be provided. Unlike supervised algorithms, unsupervised algorithms can achieve DD by relying only on structurally untagged, undamaged data. The GAN algorithm can also alleviate the lack of data diversity and class imbalance by generating data. However, there are still some difficulties to be faced. Unsupervised algorithms perform poorly on new impairments and structures, which must be solved in the future. In addition, 3D simulation tools and the Unreal Engine can also be used to make up for the lack of data. Based on ensuring the performance of the DL model, this image synthesis method can be further studied in the future.

(2) Research on data recovery methods to deal with data loss

The method to deal with data loss is data recovery. The current recovery algorithms are mainly divided into three categories, including the interpolation method based on the statistical method, ML, and DL method [128]. Considering the current big data characteristics of SHM systems, DL has higher applicability in data recovery. For example, Tang et al. [259] used the combined method of compressed sensing and CNN to achieve a good recovery effect in the case of continuous data missing. Ju et al. [73] proposed a data recovery framework based on the GRU and time correlation to improve prediction accuracy through forward and backward bidirectional prediction. Although DL has made some progress in data recovery, the problems of high-frequency components, noise interference, and random data loss problems need to be further solved.

(3) Research on anomaly detection methods to improve data quality

There will be abnormal data in the acquired data, affecting the data quality. The current research can detect abnormal data through the DL algorithm and complete data cleaning, such as CNN, LSTM, etc. However, this part of the research still needs a comparison of multiple methods, and it needs to be clarified which way can achieve better results and apply to all types of anomalies. It means this is an issue that needs attention in the future.

#### 6.2.2. Trends in Algorithms

(1) Research on model uncertainty to enhance the credibility of the results

In order to avoid the possibility of errors in applying DL in SHM, a feasible approach is to add uncertainty to the model, that is, to measure the model’s confidence. There is individual literature within the study that incorporates Bayesian inference into DL models [144], where the prediction results are output along with uncertainty. It is beneficial to decide whether to adopt the prediction results or not. The future focus should be on making uncertainty metrics more widespread and influential.

(2) Research on explainable artificial intelligence to deal with the black box characteristic

In order to better explain DL, a new research direction, namely “explainable artificial intelligence”, has received more and more attention [256]. Its purpose is to help humans understand why machines make decisions and whether they can be trusted. Angelov et al. [260] briefly analyzed the explainability of artificial intelligence, explaining the four principles of explainability and the main challenges. However, at present, the research is mainly concentrated in the medical field, and the research in the field of SHM needs to be further promoted.

#### 6.2.3. Trends in Applications

(1) Research on low-cost sensors and self-powered sensors to reduce costs in SHM

One solution for SHM system cost is to strive for advances in low-cost sensor technology so that low-cost sensors can be used widely as soon as possible. Even the transfer of sensors into intelligent devices in the structure can reduce costs. In addition, the self-powered sensor obtains energy from the structure’s vibration [55], thus avoiding the data loss caused by power interruption. This self-powered sensor should be further studied and popularized in the future.

(2) Research on smartphones and UAVs to facilitate SHM applications

Nowadays, smartphones have become a data collection tool. On the one hand, it can be used to shoot images. On the other hand, sensors such as accelerometers can be installed in smartphones as a portable method for collecting data. Smartphones are now becoming a necessary communication, social, learning, and payment platform. Associating SHM with smartphones is a forward-looking move. In the future, it will be possible to transfer DL models to cell phones and see the analytical assessment of structural conditions on the phone. Next, there is also the development of drones equipped with cameras, giving more research at remote control distance and positioning, making it an excellent method for collecting images remotely.

(3) Research on transfer learning and construction of benchmark datasets to deal with the unification problem

Transfer learning can enable DL to handle multiple tasks, with damage identification, localization, and assessment achieved by switching. The feasibility and universality of applying DL in SHM in various structures and types of damage need to be further explored. In the future, it is necessary to solve the inconsistency of datasets, labels, and experimental structures and establish a wider and more diverse benchmark dataset. The unrestricted use of the benchmark dataset enables researchers to communicate with each other and promote the development of SHM methods. It even considers setting standard levels, steps, and rules for implementing specific ways to achieve uniformity in research methods.

(4) Research on the application of wireless sensors and IoT to SHM systems

The wireless sensor has its unique advantages. Firstly, it could avoid complex wiring, which is more convenient and economical for installation and maintenance. When collecting data, wireless transmission technology can be used to realize data transmission. On the other hand, the Internet of Things (IoT) collects, transmits, and processes data remotely through wireless sensors and cloud computing—comprehensive monitoring of a building structure for digital and information management. The IoT is currently developing rapidly and bringing significant changes in SHM. Coupled with the application of DL, it will expand its possibilities and become the future development trend.

#### 6.2.4. SHMDT Architecture

Michael Grieves defined DT as a virtual, digital equivalent of a physical product [261] and proposed a three-dimensional model of DT in 2014 [262]. On this basis, Tao et al. [263] proposed the DT five-dimensional model, which includes five parts: physical entity, virtual entity, DT data, services, and connection. It is the basic conceptual model for the application of DT technology. DT, a new data-driven paradigm, can comprehensively and organically integrate physical structures, monitoring data, virtual models, data processing, and service systems. SHM acquires monitoring data by sensing physical structures through sensors and realizes service functions such as DD through data processing. There is a strong coupling between SHM and DT, with similar architectures and core elements. We conclude that SHM will develop in the direction of the DT system. Therefore, we consider constructing the SHMDT model to make SHM develop towards digitalization, integration, intelligence, and visualization.

**Figure 17:**
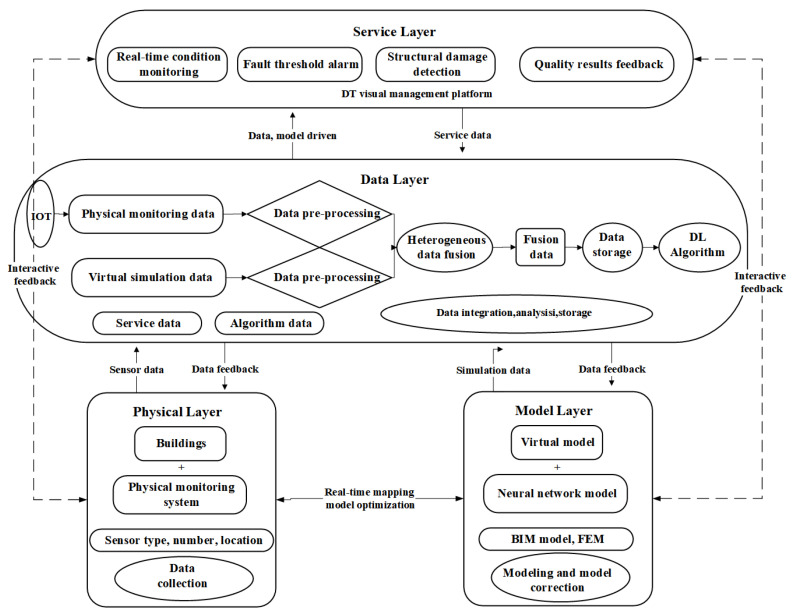
SHMDT architecture diagram.

As shown in Figure 17, The SHMDT model is based on the DT five-dimensional model. Real-time loads and responses are collected by installing sensors in critical parts of the physical layer building through a physical monitoring system. The model layer contains a virtual model of the building updated using real-time data, which can generate simulation data. The data layer combines physical monitoring, virtual simulation, service, and algorithm data. The monitoring and simulation data are merged using heterogeneous data fusion methods to form fused data stored in the database, which is subsequently applied to drive DL algorithms for structural DD. The service layer implements real-time condition monitoring, fault threshold alarm, structural damage detection, and quality result feedback by constructing a DT visual management platform. Users can achieve digital, integrated, intelligent, and visual management through a software platform. The transmission layer is the channel to realize the information interaction and feedback between the layers. The real-time and accuracy of information collection and transmission are ensured using wireless communication technology. In developing SHMDT models, critical technologies such as the IoT, model simulation, heterogeneous data fusion, data storage, DL algorithms, big data analysis, cloud computing, and DT software platforms (Unity3D2021.2.10f1c1 Personal) must be combined.

## 7. Conclusions

This paper researches DL applications in SHM following the sequence of data collection, the DL method, the application object, and the application function. Each chapter relies on a summary table of the retrieved literature, for which a specific analysis is carried out, and the corresponding rules are summarized.

- The data and collection methods of DL applied in SHM are analyzed statistically. The application scope and advantages and disadvantages of different data types are further analyzed on this basis. Secondly, various data acquisition methods are compared based on time consumption, cost, and data acquisition accuracy. In terms of data, vibration signals, images, acoustic emission signals, and GW signals are the most common data types, of which vibration signals account for the highest proportion of research, suitable for detecting various damage. Sensors and cameras are the most direct and commonly used tools for data acquisition. In addition, the rapid development of drones and smartphones have also become popular image acquisition tools.
- The statistical analysis of the DL method used by DL in SHM is carried out. Firstly, the architecture and application range of DL algorithms such as the CNN, RNN, auto-encoder, and GAN are introduced. Next, we further summarize the data types, core functions, and applications applicable to the different DL methods. At the same time, the data scale involved in the DL algorithm is clarified by enumerating the size and nature of some literature datasets. The last part shows the literature comparing different DL algorithm performances.
- The application object and function of DL in SHM are analyzed statistically. This paper divides the application objects into two categories: facilities and facilities components. Facilities include bridges, frame structures, buildings, etc., and facilities components include concrete blocks, metal plates, beams, etc. Among them, the research on bridges accounts for the highest proportion. Regarding application function, the most common injuries reported in the literature include cracks, stiffness reduction, bolt loosening, support damage, and simulated damage. In addition, data loss and anomalies are also becoming a concern for researchers. The research stage can be divided into the experimental and practical application stages. Only some literature selects research objects and data from actual structures, mainly bridge databases such as Yonghe Bridge in Tianjin, Saigon Bridge in Vietnam, and bridge inspection records in South Korea.
- Challenges and trends are identified in part based on bibliometric and literature analysis. First, we identify the challenges of applying DL to SHM from three levels: data, algorithm, and application. The issues involved include the lack of data, uncertainty of the model algorithm, application cost, etc. Given the challenges, we put forward the corresponding solutions in the trend section and as a future research direction for reference. For example, researchers can focus on the FE, unsupervised algorithms, data recovery, self-powered sensors, IoT, and other research directions in the future. Moreover, SHM can be combined with the current research focus on DT to build a five-dimensional model framework of SHMDT, which supports SHM development in integration, intelligence, digitalization, and visualization.

This paper also has some limitations. Firstly, regarding research articles, it focuses more on CNNs and is only retrieved through the WOS search platform. Secondly, the article has three perspectives: data, DL algorithm, and application. Each perspective contains a wide range of content, leading to some detailed content not being discussed thoroughly. In the future, the research scope will be expanded to conduct more extensive and in-depth research on the application of DL in SHM.

## Figures and Tables

**Table 1:** Comparison of different data types.

Data Type	Damaged Type	Data Acquisition Key Points	Data Processing Key Points	Pros and Cons of Application
Vibration signal	Various damages	Sensors and noise	Time-varying characteristics	Insensitive to minor injuries
Image	Surface damages	Shooting equipment and environment	Computational complexity	Damage visualization
AE signal	AE source location	Sensors and noise	AE signal uncertainty	Dependence on the laboratory environment
GW signal	Metal structure damages	Sensors and noise	GW signal multi-modal	Sensitive to minor injuries

**Table 2:** Comparison of different acquisition methods.

Acquisition Method	Time Spent	Cost	Data Accuracy
Sensor	Long-term	Relatively low	High
FE simulation	Medium-term	Relatively high	Moderate
Public dataset	Short-term	Low	Moderate
Online searching	Short-term	Low	Moderate
Camera	Medium-term	High	High
Video camera	Medium-term	High	High
Mobile phone	Medium-term	Moderate	Moderate
UAV	Medium-term	High	High

**Table 3:** Comparison of different algorithm types.

Algorithm Type	Data Type	Core Function	Applications Function	Pros and Cons
CNN	Images, Time series data	Object recognition Image classification Semantic segmentation	Crack, corrosion, stiffness reduction, support failure, bolt loosening, displacement, stress, delamination detection, denoising, data recovery	It extracts high-level features but suffers from overfitting and data dependence.
RNN	Time series data	Object recognition Sequence identification	Stiffness reduction, displacement, delamination detection, data anomaly detection	It is good at capturing time sequence information but has the problem of disappearing gradients.
Auto-encoder	Images, Time series data	Object recognition Semantic segmentation Data enhancement	Crack, erosion, cable tension, stiffness, displacement detection, AE source location Data anomaly detection	It is good at dimensionality reduction of data similar to training samples
GAN	Images, Time series data	Object recognition Image classification Semantic segmentation Data enhancement	Crack, spalling detection, Image generation Missing data interpolation	It can be used to generate images, but training is unstable.

**Table 4:** Example of a data case applying DL to SHM.

Reference(s)	Function	Data Type	Data Set	Percentage of Training Set and Test Set
Xiao et al. [16]	Transfer learning in bridge damage diagnosis	Vibration signal	Three datasets, each containing 2800 samples	1:1
Coraca et al. [200]	Bearing damage and cable slack detection	Vibration signal	2085 undamaged and 3184 damaged samples	1587:828:769
Fathnejat et al. [110]	Combination of 1D-CNN and RNN for damage detection	Vibration signal	IASC-ASCE benchmark model with nine scenarios under each scenario with an input matrix dimension of 72,000 × 16	6:2:2
He et al. [17]	Aluminum frame stiffness reduction identification	Vibration signal	A total of 13,140 acceleration samples for the four stiffness states	6:2:2
Jiang et al. [128]	Recovery of random data loss	Vibration signal	128 five-minute sample segments, 38,144 actual acceleration samples	8:1:1
Guo et al. [9]	Acoustic emission-based impact source localization problem	AE	500 good single-channel signals and 100 bad signals that are poorly clamped, loosely connected, etc.	9:1
Ebrahimkhanlou et al. [81]	Localization of AE sources from metal plates	AE	Acoustic emission signals collected from 576 analog sources	8:1:1
Liao et al. [22]	Damage localization in composite structures	GW	The Gramian angular field was used to convert GW signals into 2D images, resulting in 10,620 128 × 128 × 3pixel images	7:2:1
Lomazzi et al. [201]	Localization and quantification of cracks in aluminum panels	GW	100 damaged Lamb wave replicas and 51,600 undamaged Lamb wave replicas	70:28:2
Sawant et al. [23]	Temperature-compensated damage identification and localization	GW	Generated 16,000 samples based on the OGW dataset with added noise	70:24:6
Kao et al. [36]	Identification and quantification of bridge cracks	Image	1463 mobile phone images and 3006 SDNET dataset images	8:2
Kulkarni et al. [202]	Pavement void inspection	Image	4900 principal component thermography and sparse principal component thermography maps	3920:980
Lu et al. [47]	Loose Bolt Detection	Image	300 cell phone captured images, 1600 composite images	4:1:1 (Real datasets); 6:1:1 (Synthetic datasets)
Panta et al. [39]	Pixel-level detection of cracks in dams	Image	Dam crack image 1650, extended image 101, DeepCrack dataset 237, and enhanced image	_
Wang et al. [44]	Crack localization and assessment	Image	2177 small images with a resolution of 500 × 500	7:2:1
Zhao et al. [54]	Concrete dam void, spalling cracks, and six other types of damage detection	Image	2500 images of 640×640pixel resolution	6:2:2
Dunphy et al. [84]	Detection of multiple types of damage to concrete structures	Image	Extracted 5115 images from the SDNET2018 dataset, containing 2450 undamaged images and 2665 damaged images	7:2:1
Li et al. [48]	Detection of surface defects in welded steel bars	Image	1580 images with a resolution of 3204 × 4032 taken by a cell phone	7:1:2
Song et al. [51]	Measurement of structural displacement using computer vision	Image	300 images decomposed from video	180:120
Choi et al. [203]	Segmentation of concrete cracks in the image	Image	200 images of different pixel resolutions were collected through mobile phones and the Internet	4:1

**Table 5:** Example of DL method comparison.

Reference(s)	Goal	DL Algorithm(s)	Effect		
Wang et al. [44]	Image-based crack location and evaluation	YOLOX	88.5%		MAP
		Faster R-CNN	69.77%		
		Deconvolutional Single Shot Detector	86%		
		YOLOv5	86%		
Li et al. [38]	Image-based inspection of bridge bolts, nuts, and nut holes	CNN	95.6%		Accuracy
		LSTM	93%		
		YOLOv4	76.5%		
Tang et al. [204]	Bridge weight and speed identification based on random response power spectral density	AlexNet	96%		Accuracy
		VGG16	96%		
		InceptionV3	96.75%		
		ResNet50	93%		
Arafin et al. [132]	Image-based classification of multiple defects on concrete surfaces		Accuracy	Precision	Recall
		IncptionV3	91%	83%	100%
		Xception	90%	81%	100%
		MobileNetV2	82%	71%	94%
		ResNet50	82%	69%	89%
		VGG19	61%	64%	80%
Ijjeh et al. [205]	Layered detection and localization of composite materials based on full wave field measurements		Accuracy	Precision	Recall
		Res-UNet	99.4%	98.9%	100%
		VGG16 encoder–decoder	99.1%	98.1%	100%
		FCN-DenseNet	99.7%	99.4%	100%
		PSPNet	98.4%	96.8%	100%
		Global Convolutional Network	100%	100%	100%

## Data Availability

Not applicable.

## Supplementary Materials

The following supporting information can be downloaded at https://www.mdpi.com/article/10.3390/s23218824/s1, Table S1: Data and access summary table. Table S2: DL algorithm summary table. Table S3: Facility component application summary table. Table S4: Facility application summary table. Table S5: Other application functions summary table.

## Abbreviations

1D-CNN	One-Dimensional Convolutional Neural Networks
2D-CNN	Two-Dimensional Convolutional Neural Networks
AE	Acoustic Emission
ANN	Artificial Neural Network
ASCE	American Society of Civil Engineers
BPTT	Back-Propagation Through Time
BSHM	Bridge Structure Health Monitoring
BWIM	Bridge Weigh-In-Motion
CFRP	Carbon Fiber-Reinforced Polymer
CNN	Convolutional Neural Network
CWT	Continuous Wavelet Transform
DCGAN	Deep Convolutional GANs
DCNN	Deep Convolutional Neural Network
DD	Damage Detection
DGBO	Deep Generative Bayesian Optimization
DL	Deep Learning
DNNs	Deep Neural Networks
DT	Digital Twin
EMI	Electro-Mechanical Impedance
FCN	Fully Convolutional Networks
FEM	Finite Element Model
GAN	Generative Adversarial Networks
GNN	Graph Neural Network
GPR	Ground Penetrating Radar
GRU	Gated Recurrent Unit
GW	Guided Wave
IASC	International Association for Structural Control
IoT	Internet of Things
LSTM	Long Short-Term Memory
MAP	Mean Average Precision
Mask R-CNN	Mask Region-Based Convolutional Neural Networks
METU	Middle East Technical University
ML	Machine Learning
MLP	Multi-Layer Perceptron
NDT	Non-Destructive Testing
PZT	Piezoelectric Transducer
QUGS	Qatar University Grandstand Simulator
R-CNN	Region-based Convolutional Neural Networks
RNN	Recurrent Neural Networks
SHM	Structural Health Monitoring
SHMDT	Structural Health Monitoring Digital Twin
SSD	Single Shot MultiBox Detector
TCRF	Continuous Rigid Frame Bridge
UAVs	Unmanned Air Vehicles
UGW	Ultrasonic Guided Wave
YOLO	You Look Only Once
