# Supplementary material for: Deep Learning for Structural Health Monitoring: Data, Algorithms, Applications, Challenges, and Trends
Source: Sensors (Basel). 2023 Oct 30;23(21):8824. doi: 10.3390/s23218824 (PMC10650096; doi:10.3390/s23218824)
Supplement: Supplementary file 1 [file sensors-23-08824-s001.zip › sensors-2678843-supplementary.pdf]

SENSORS

Deep learning for structural health monitoring: data, algorithms, applications, challenges, and trends

Jing Jia 1, Ying Li 2,*

[1] Department of Civil Engineering, College of Engineering, Ocean University of China, Qingdao 266100, China; jingjia@ouc.edu.cn.

[2] Department of Civil Engineering, College of Engineering, Ocean University of China, Qingdao 266100, China; liying4555@stu.ouc.edu.cn.

*Correspondence should be addressed to Ying Li; liying4555@stu.ouc.edu.cn

Data

Table S1. Data and access summary table

Data type	Quantity percentage	Acquisition method	Articles
Vibration signal(acceleration, displacement, strain)	52.7%	Acceleration sensor	[1-71]
		Strain sensor	[6, 23, 54, 72-92]
		Displacement sensor	[6, 66, 79-81, 89, 93-96]
		Numerical simulation (finite element)	[1-4, 6-8, 10, 11, 15, 16, 20, 21, 23, 31, 40, 47, 49, 56, 60-62, 64, 70, 74, 79, 80, 92, 97-133]
		Public dataset	[5, 16, 55, 57, 99, 134-154]
		SHM system acquisition	[155-161]
		Motion capture system (MCS)	[105]

		Measured by plumb lines (PL) and inverted plumb lines (IP)	[162]
Image	26.74%	Camera	[101, 108, 163-200]
		Mobile phone	[172, 193, 201-209]
		Unmanned air vehicle	[174, 207, 210-215]
		Traffic camera	[216-220]
		Online Searching	[163, 168, 172, 190, 197, 219, 221-223]
		Public dataset	[190, 192, 207, 215, 224-245]
		GAN generation	[246, 247]
		Infrared camera	[248]
		Simulation tool	[164, 209]
Video	0.77%	Video camera	[249-251]
Acoustic emission	3.34%	AE sensor	[252-263]
		PZT sensor	[264]
Guided wave	6.68%	PZT sensor	[265-280]
		Numerical simulation (finite element)	[271, 278, 281-286]
		Public dataset	[287]

		AE sensor	[288]
Electromechanical impedance	2.06%	PZT sensor	[289-295]
		Numerical simulation (finite element)	[293]
Ground penetrating radar data	0.26%	Public dataset	[296]
Tension data	0.77%	Cable dynamometer	[297]
		Program simulation	[298]
		Competition projects provided	[299]
Temperature data	2.57%	Temperature sensor	[24, 30, 93, 300-304]
		Weather station provided	[83]
		Fiber	[305]
Wind speed	1.54%	Anemometer	[24, 82, 303, 304, 306]
		Numerical simulation (finite element)	[307]
Impact signal	0.26%	PVDF (Poly (vinylidene fluoride)) ribbon sensors	[308]
Basic data of bridges	0.26%	National Bridge Inventory (NBI) database	[309]
Concrete deck condition index	0.26%	Bridge inspection history database	[310]
Electrical resistance	0.26%	Digital multimeter	[311]

Vehicle load	0.77%	Weigh-in-motion sensor	[24, 301, 303]
Power spectral density	0.26%	Numerical simulation (finite element)	[312]
Acoustic data	0.26%	Geophones	[313]
Electrical Resistance Tomography (ERT)	0.26%	Numerical simulation (finite element)	[314]

Algorithm

Table S2. DL algorithm summary table

Algorithm type	Quantity percentage	Specific Algorithm	Articles
CNN	65.59%	1D-CNN	[1-3, 16, 20, 21, 28, 41, 54, 58, 59, 69, 71, 74, 88, 99, 100, 102, 107, 128, 130, 138, 141, 143, 144, 147, 153, 213, 259, 268, 270, 271, 278, 295, 297]
		2D-CNN	[6, 7, 14, 23, 25, 33, 40, 42, 43, 45, 51-53, 56, 60, 66, 67, 72, 78-80, 82, 83, 85, 87, 88, 92, 95-99, 101, 103, 105, 108, 109, 115, 117, 118, 122, 124, 125, 132, 139-141, 146, 155, 157, 158, 168, 185, 194, 222, 225, 232, 247, 249, 253, 256, 259, 260, 262, 267, 272-276, 282-285, 288-290, 304, 308, 309, 311]
		3D-CNN	[188]
		LeNet-5	[224]
		DCNN	[9, 26, 36, 73, 116, 142, 173, 200, 210, 211, 215, 228, 242, 265, 266, 286, 292, 293, 312]
		AlexNet	[63, 195, 219, 227, 258]
		VGG	[172, 187, 192, 219, 239, 243, 261]
		GoogleNet	[248, 252]

		ResNet	[10, 120, 156, 199, 223, 239, 240, 263, 291, 296]
		R-CNN	[163, 164]
		Faster R-CNN	[163, 198, 201, 219]
		YOLO	[177, 205, 207-209, 214, 216, 217, 220, 229]
		SSD	[203]
		FCN	[30, 104, 131, 133, 149, 166, 167, 184, 190, 193, 196, 204, 212, 226, 233-236, 281]
		U-net	[165, 180, 191, 193, 221, 226, 238, 306]
		DenseNet	[31, 47, 231]
		Deeplabv3+	[120, 171, 193, 231]
		Mask R-CNN	[170, 189, 197, 202, 206, 218, 230]
		Flownet	[169]
		FDCNN	[38, 39]
		Hierarchical CNN	[114]
Hybrid algorithm	4.71%	CNN+GRU	[55, 57, 159]
		CNN+LSTM	[27, 70, 186, 294, 302, 305, 307]
		CNN+RNN	[145]

		CNN+LSTM+Yolov4	[181]
		CNN+Auto-encoder	[119]
		Deeplabv3+Class activation map	[178]
		CNN+ESN	[5]
		CNN+Ensemble Empirical Mode Decomposition (EEMD)+Pearson Correlation Coefficient (PCC),	[18]
RNN	8.82%	Traditional RNN	[93, 136]
		LSTM	[12, 32, 35, 48, 65, 75, 76, 81, 89, 94, 99, 137, 151, 160, 162, 254, 256, 264, 271, 287, 299, 300, 310]
		GRU	[34, 50, 86, 121, 301]
Auto-encoder	8.53%	Auto-encoder	[15, 24, 44, 46, 49, 59, 182, 183, 303, 313]
		Stacked auto-encoder	[77, 127, 252, 255, 260, 267]
		Sparse auto-encoder	[8, 11]
		Variational auto-encoder	[17, 61, 64]
		Convolutional auto-encoder	[33, 123, 154, 265, 277]
		Denoising auto-encoder	[110, 111]
		Stacked denoising auto-encoder	[257]

GAN	2.94%		[62, 84, 89, 129, 134, 152, 179, 237, 244, 246]
Other algorithms	9.41%	MLP	[22, 99, 110, 271]
		DNN	[13, 68, 112]
		ANN	[19, 280, 314]
		CapsNets	[4]
		Transformer	[91, 106, 176, 279]
		GNN	[126, 269, 298]
		Deep convolutional image-denoiser networks	[161]
		Deep principal component analysis	[135]
		EfficientDet	[174]
		Magicpoint	[251]
		Learning-based model for video motion magnification	[250]
		Channel-spatial-temporal attention-based network	[148]
		Trident	[113]
		HRNet	[241]

	Siamese neural network	[29]
	Temporal-Spatial-Load (TSL) model	[90]
	Dynamic data fusion convolution	[150]
	Semantic damage detection network	[245]
	Deep learning network	[37]
	Dense pose object detector	[175]

Application

Table S3. Facility component application summary table

Facility component types	Quantity percentage	Application functions	Articles	Application results
Concrete block	7.14%	Crack	[248, 254]	Identification
		Bughole	[173]	Identification
		Displacement	[202, 204]	Location
		ASR	[252]	Identification
		Carbonation	[136]	Identification
Reinforced concrete block	2.04%	Bond strength	[294]	Assessment
		Stress monitoring	[282]	Assessment

Composite plate	16.33%	Delamination	[88, 281, 286, 287, 308]	Identification, Location
		Crack	[264, 265, 275]	Location, Assessment
		Simulated damage (paste block)	[271, 272, 277, 311]	Location
		Artificial AE source	[253]	Location
		FE elastic modulus decreases	[103]	Location, Assessment
		Impact	[279]	Identification
		Hole	[120]	Assessment
Aluminum plate	25.51%	Crack	[258, 264, 265, 273, 276, 278, 283, 285]	Assessment
		Simulated damage	[268, 280, 289, 290]	Identification
			[270, 280]	Location
			[266]	Location, Assessment
		Artificial AE source	[259]	Identification
			[255, 260]	Location
		FE elastic modulus decreases	[106]	Assessment
		Bughole	[278, 293]	Location, Assessment
		Corrosion	[267, 278]	Location, Assessment

		Impact	[263]	Location
		Stress Monitoring	[274]	Assessment
Steel plate	5.10%	Crack	[101]	Assessment
			[109]	Identification, Location
		Simulated damage	[269]	Location, Assessment
		Artificial AE source	[256, 257]	Location
Steel beam	20.41%	Crack	[77, 85, 233]	Identification
			[52, 167, 291, 292]	Location, Assessment
			[100, 101]	Assessment
		Simulated damage	[2]	Identification
		Stiffness degradation	[4, 30, 31, 107]	Location, Assessment
		Notch	[20]	Identification
		Displacement	[72, 184, 187, 249, 251]	Assessment
Concrete beam	2.04%	Crack	[236]	Identification
		Stiffness degradation	[99]	Assessment
Reinforced concrete beam	6.12%	Crack	[179, 185]	Identification, Assessment

		Structural drift	[199]	Assessment
		Prestress	[295]	Assessment
		Young 's modulus decreases	[8, 11]	Assessment
Simple beam	2.04%	Stiffness degradation	[73]	Assessment
		Stiffness degradation	[102]	Location
Aluminum beam	2.04%	Stiffness degradation	[119]	Identification
		Simulated damage	[36]	Identification
Truss structure	2.04%	Bolt looseness	[1]	Identification
		Brace damage	[21]	Identification
Steel pipeline	3.06%	Crack	[19, 225]	Identification
		Notch	[284]	Identification
Mat foundation	1.02%	Crack	[262]	Identification
Masonry wall	2.04%	Crack	[168, 180]	Identification
Concrete panel	1.02%	Crack	[195]	Identification
Iron plate	1.02%	Bolt looseness	[209]	Identification
Concrete plate	1.02%	Crack	[177]	Identification, Location, Assessment

Table S4. Facility application summary table

Facility types	Quantity percentage	Application functions	Articles	Application results
Bridge	38.5%	Cracks	[55, 57, 163, 193, 213, 241, 243, 247]	Identification
			[61]	Location
			[5, 10, 67, 76, 194]	Assessment
			[15, 211, 231]	Identification, Location
			[134, 207]	Identification, Location, Assessment
		Steel bar exposure / fracture	[66, 228, 235]	Identification
			[296]	Location
		Stiffness degradation	[56, 104, 122]	Location
			[8, 11, 16, 44, 112, 113, 135, 309]	Assessment
		Rupture of tendons, pier settlement	[147, 151, 153, 154]	Assessment
			[134]	Identification, Location, Assessment
		Cable tension reduction	[99]	Identification, Location, Assessment
			[70, 171, 212, 297]	Identification, Assessment
			[298]	Location, Assessment

		Vehicle-related	[63, 216-220]	Weight assessment
			[130, 312]	Vehicle Identification
		Material deterioration	[60, 115, 310]	Assessment
		Displacement	[23, 93, 169, 182, 251, 303]	Assessment
		Deflection	[301, 302]	Prediction
		Deformation	[188]	Identification
		Bolt looseness	[164]	Identification, Assessment
		FE elastic modulus decreases	[97]	Location
		Strain	[87, 91, 304, 305]	Prediction
		Nuts, Bolts, Nut holes	[181]	Identification
		FE elastic modulus decreases	[118]	Identification
		Vortex-induced vibrations	[24]	Prediction
		Simulated damage	[25, 26, 124]	Identification, Location, Assessment
		Driving segment	[27]	Identification
		Crowd	[54]	Weight assessment
Steel frame	22.5%	Bolt looseness, Brace damage	[5, 7, 55, 57, 71, 140-143]	Assessment

			[145, 146, 148, 150, 152]	Identification
		Brace damage	[69, 126, 132, 147, 250]	Identification
		Bolt looseness	[9, 13, 144, 201]	Identification
			[203]	Identification, Location
			[3, 78]	Assessment
			[134, 154]	Assessment, Location
		Stiffness degradation	[10, 40, 47, 49, 70, 98, 99, 127]	Assessment
			[95, 104, 105]	Location
		Displacement	[51, 117, 128, 208]	Post-earthquake safety assessment
			[123, 175]	Assessment
Reinforced concrete frame	1.00%	Deformation	[139]	Location, Assessment
		Displacement	[133]	Location, Assessment
Aluminum frame	1.50%	Displacement	[38, 39]	Assessment
		Stiffness degradation	[18]	Assessment
Buildings	9.00%	Cracks	[189]	Identification, Location, Assessment
			[165, 205, 210, 224, 239, 245]	Identification

		Displacement	[96, 249]	Assessment
			[43]	Post-earthquake safety assessment
		Mould, Deterioration, Stain	[172]	Identification
		Load	[46]	Assessment
		Steel surface defects	[183]	Identification
		Stiffness degradation	[28, 37]	Identification
			[35]	Assessment
		Modal response	[32]	Characterization
		Acceleration	[307]	Prediction
Pavement	2.50%	Cracks, Visco-plastic deformation, Surface defects	[206]	Identification, Location, Assessment
		Humps, Manhole covers, Potholes	[68]	Identification
		Cracks	[176, 231]	Identification
		Voids	[174]	Identification
Rail	3.00%	Rail fastener	[131]	Assessment
		Cracks, Squats, Corrugations, Rust	[232]	Identification, Location
		Railway mechanical state	[186]	Assessment

		Cracks	[186, 261, 288]	Assessment
Tunnel	5.00%	Cracks	[170, 191, 240]	Identification
			[196, 198]	Identification, Location
		Cracks, Leakage	[166, 178]	Identification, Location
		Mechanical behavior	[90]	Prediction
		Stiffness degradation	[111]	Location, Assessment
		Tether stiffness degradation	[125]	Identification
Dam	3.50%	Displacement	[94, 159, 162, 300]	Assessment
		Precipitates, Voids and Pits, Spalling, Cracks, Water stains, Concrete stones.	[214]	Identification
		Erosion	[313]	Identification
		Crack	[238]	Identification
Hydropower station	0.50%	Cracks	[200]	Identification
Offshore platform	1.50%	Brace damage	[74]	Location, Assessment
		Crack bar	[29]	Identification
		Topside mass	[110]	Assessment
Offshore wind turbine	1.50%	Tendon damage	[116]	Identification

		Acceleration, Tower root force	[22]	Identification
Offshore wind turbine jacket foundations	0.50%	Cracks, Bolt looseness	[42]	Identification
Gymnasium	0.50%	Fastener defects	[114]	Identification
Transmission tower	0.50%	Fastener defects	[17]	Identification, Location
Other infrastructure	9.00%	Cracks	[190, 197, 215, 221, 223, 227, 237, 244, 314]	Identification
			[222, 234]	Identification, Location
			[53, 192, 226, 229, 246]	Assessment
		Spalling	[242]	Identification
		Corrosion, Cracks, Fastener defects	[230]	Identification

Table S5. Other application functions summary table

Application functions	Quantity percentage	Articles	Application results
Data anomalies	34.21%	[12, 33, 58, 59, 65, 86, 121, 137, 138, 155, 157, 158, 299]	Anomaly detection
Data loss	57.89%	[6, 14, 34, 41, 45, 48, 50, 62, 75, 79-84, 89, 92, 108, 129, 149, 160, 306]	Data recovery
Sensor placement	2.63%	[64]	Optimal sensor placement
Noise	5.26%	[156, 161]	Noise reduction

1. Seventekidis, P. and D. Giagopoulos. A combined finite element and hierarchical Deep learning approach for structural health monitoring: Test on a pin-joint composite truss structure. Mechanical Systems and Signal Processing **2021**, 157, 23.
2. Seventekidis, P.; D. Giagopoulos; A. Arailopoulos, and O. Markogiannaki. Structural Health Monitoring using deep learning with optimal finite element model generated data. Mechanical Systems and Signal Processing **2020**, 145, 21.
3. Sharma, S. and S. Sen. One-dimensional convolutional neural network-based damage detection in structural joints. Journal of Civil Structural Health Monitoring **2020**, 10, 1057-1072.
4. Barraza, J.F.; E.L. Droguett; V.M. Naranjo, and M.R. Martins. Capsule Neural Networks for structural damage localization and quantification using transmissibility data. Applied Soft Computing **2020**, 97,
5. He, Y.Y.; L.K. Zhang; Z.S. Chen, and C.Y. Li. A framework of structural damage detection for civil structures using a combined multi-scale convolutional neural network and echo state network. Engineering with Computers **2023**, 39, 1771-1789.
6. Park, H.S.; J.H. An; Y.J. Park, and B.K. Oh. Convolutional neural network-based safety evaluation method for structures with dynamic responses. Expert Systems with Applications **2020**, 158, 14.
7. Teng, Z.Q.; S. Teng; J.Q. Zhang; G.F. Chen, and F.S. Cui. Structural Damage Detection Based on Real-Time Vibration Signal and Convolutional Neural Network. Applied Sciences-Basel **2020**, 10, 15.
8. Finotti, R.P.; F.D. Barbosa; A.A. Cury, and R.L. Pimentel. Novelty Detection Using Sparse Auto-Encoders to Characterize Structural Vibration Responses. Arabian Journal for Science and Engineering **2022**, 47, 13049-13062.
9. Zhang, T.; S. Biswal, and Y. Wang. SHMnet: Condition assessment of bolted connection with beyond human-level performance. Structural Health Monitoring-an International Journal **2020**, 19, 1188-1201.
10. Wang, R.H.; Chencho; S.J. An, et al. Deep residual network framework for structural health monitoring. Structural Health Monitoring-an International Journal **2021**, 20, 1443-1461.
11. Finotti, R.P.; F.D. Barbosa; A.A. Cury, and R.L. Pimentel. Numerical and Experimental Evaluation of Structural Changes Using Sparse Auto-Encoders and SVM Applied to Dynamic Responses. Applied Sciences-Basel **2021**, 11, 18.
12. Liu, G.; L.L. Li; L.L. Zhang; Q. Li, and S.S. Law. Sensor faults classification for SHM systems using deep learning-based method with Tsfresh features. Smart Materials and Structures **2020**, 29, 15.
13. Kohiyama, M.; K. Oka, and T. Yamashita. Detection method of unlearned pattern using support vector machine in damage classification based on deep neural network. Structural Control & Health Monitoring **2020**, 27, 23.
14. Tang, Z.Y.; Y.Q. Bao, and H. Li. Group sparsity-aware convolutional neural network for continuous missing data recovery of structural health monitoring. Structural Health Monitoring-an International Journal **2021**, 20, 1738-1759.
15. Hurtado, A.C.; K. Kaur; M.M. Alamdari, et al. Unsupervised learning-based framework for indirect structural health monitoring using adversarial autoencoder. Journal of Sound and Vibration **2023**, 550, 23.

16. Xiao, H.T.; H. Ogai, and W.J. Wang. A new deep transfer learning method for intelligent bridge damage diagnosis based on muti-channel sub-domain adaptation. Structure and Infrastructure Engineering **2023**, 16.
17. Coraca, E.M.; J.V. Ferreira, and E.G.O. Nobrega. An unsupervised structural health monitoring framework based on Variational Autoencoders and Hidden Markov Models. Reliability Engineering & System Safety **2023**, 231, 15.
18. He, Y.Y.; Z.H. Huang; D. Liu; L.K. Zhang, and Y. Liu. A Novel Structural Damage Identification Method Using a Hybrid Deep Learning Framework. Buildings **2022**, 12, 18.
19. Sandhu, H.K.; S.S. Bodda, and A. Gupta. Post-hazard condition assessment of nuclear piping-equipment systems: Novel approach to feature extraction and deep learning. International Journal of Pressure Vessels and Piping **2023**, 201, 16.
20. Wu, C.S.; Y.X. Peng; D.B. Zhuo, et al. Energy Ratio Variation-Based Structural Damage Detection Using Convolutional Neural Network. Applied Sciences-Basel **2022**, 12, 19.
21. Seventekidis, P. and D. Giagopoulos. Model error effects in supervised damage identification of structures with numerically trained classifiers. Mechanical Systems and Signal Processing **2023**, 184, 31.
22. Wang, Z.M.; D.S. Qiao; G.Q. Tang, et al. An identification method of floating wind turbine tower responses using deep learning technology in the monitoring system. Ocean Engineering **2022**, 261, 12.
23. Ni, P.; Y.X. Li; L.M. Sun, and A. Wang. Traffic-induced bridge displacement reconstruction using a physics-informed convolutional neural network. Computers & Structures **2022**, 271, 13.
24. Kim, S. and T. Kim. Machine-learning-based prediction of vortex-induced vibration in long-span bridges using limited information. Engineering Structures **2022**, 266, 15.
25. Dang, H.; M. Tatipamula, and H.X. Nguyen. Cloud-Based Digital Twinning for Structural Health Monitoring Using Deep Learning. Ieee Transactions on Industrial Informatics **2022**, 18, 3820-3830.
26. Hajializadeh, D. Deep learning-based indirect bridge damage identification system. Structural Health Monitoring-an International Journal **2023**, 22, 897-912.
27. Shin, R.; Y. Okada, and K. Yamamoto. Application of C-LSTM Networks to Automatic Labeling of Vehicle Dynamic Response Data for Bridges. Sensors **2022**, 22, 12.
28. Ragab, M.; M. Lazhari, and M.L. Nehdi. Localization and classification of structural damage using deep learning single-channel signal-based measurement. Automation in Construction **2022**, 139, 19.
29. Baquerizo, J.; C. Tutiven; B. Puruncajas; Y. Vidal, and J. Sampietro. Siamese Neural Networks for Damage Detection and Diagnosis of Jacket-Type Offshore Wind Turbine Platforms. Mathematics **2022**, 10, 20.
30. Torzoni, M.; L. Rosafalco; A. Manzoni; S. Mariani, and A. Corigliano. SHM under varying environmental conditions: an approach based on model order reduction and deep learning. Computers & Structures **2022**, 265, 20.
31. Wang, X.Y. and Y. Xia. Knowledge transfer for structural damage detection through re-weighted adversarial domain adaptation. Mechanical Systems and Signal Processing **2022**, 172, 19.
32. Gonzalez, W.M.; A. Ferrada; R.L. Boroschek, and E.L. Droguett. Characterization of the modal response using Deep recurrent neural networks. Engineering Structures **2022**, 256, 16.

33. Jana, D.; J. Patil; S. Herkal; S. Nagarajaiah, and L. Duenas-Osorio. CNN and Convolutional Autoencoder (CAE) based real-time sensor fault detection, localization, and correction. Mechanical Systems and Signal Processing **2022**, 169, 30.
34. Ju, H.W.; Y. Deng; W.Q. Zhai, and A.Q. Li. Recovery of Abnormal Data for Bridge Structural Health Monitoring Based on Deep Learning and Temporal Correlation. Sensors and Materials **2022**, 34, 4491-4505.
35. Yang, Q. and D.J. Shen. Learning Damage Representations with Sequence-to-Sequence Models. Sensors **2022**, 22, 14.
36. Mousavi, Z.; M.M. Ettefagh; M.H. Sadeghi, and S.N. Razavi. Developing deep neural network for damage detection of beam-like structures using dynamic response based on FE model and real healthy state. Applied Acoustics **2020**, 168, 17.
37. Zhou, C.; J.G. Chase, and G.W. Rodgers. Degradation evaluation of lateral story stiffness using HLA-based deep learning networks. Advanced Engineering Informatics **2019**, 39, 259-268.
38. Lopez-Pacheco, M.; J. Morales-Valdez, and W. Yu. Frequency domain CNN and dissipated energy approach for damage detection in building structures. Soft Computing **2020**, 24, 15821-15840.
39. Morales-Valdez, J.; M. Lopez-Pacheco, and W. Yu. Automated damage location for building structures using the hysteretic model and frequency domain neural networks. Structural Control & Health Monitoring **2020**, 27, 15.
40. Ghahremani, B.; M. Bitaraf; A.K. Ghorbani-Tanha, and R. Fallahi. Structural damage identification based on fast S-transform and convolutional neural networks. Structures **2021**, 29, 1199-1209.
41. Fan, G.; J. Li, and H. Hao. Lost data recovery for structural health monitoring based on convolutional neural networks. Structural Control & Health Monitoring **2019**, 26, 21.
42. Puruncajas, B.; Y. Vidal, and C. Tutiven. Vibration-Response-Only Structural Health Monitoring for Offshore Wind Turbine Jacket Foundations via Convolutional Neural Networks. Sensors **2020**, 20, 19.
43. Alcantara, E.A.M.; M.D. Bong, and T. Saito. Structural Response Prediction for Damage Identification Using Wavelet Spectra in Convolutional Neural Network. Sensors **2021**, 21, 22.
44. Sarwar, M.Z. and D. Cantero. Deep autoencoder architecture for bridge damage assessment using responses from several vehicles. Engineering Structures **2021**, 246, 16.
45. Sun, S.B.; Y.Y. He; S.D. Zhou, and Z.J. Yue. A Data-Driven Response Virtual Sensor Technique with Partial Vibration Measurements Using Convolutional Neural Network. Sensors **2017**, 17, 22.
46. Rosafalco, L.; A. Manzoni; S. Mariani, and A. Corigliano. An Autoencoder-Based Deep Learning Approach for Load Identification in Structural Dynamics. Sensors **2021**, 21, 32.
47. Wang, R.H.; J. Li; Chencho, et al. Densely connected convolutional networks for vibration based structural damage identification. Engineering Structures **2021**, 245, 14.
48. Li, L.C.; H.J. Zhou; H.N. Liu; C.D. Zhang, and J.H. Liu. A hybrid method coupling empirical mode decomposition and a long short-term memory network to predict missing measured signal data of SHM systems. Structural Health Monitoring-an International Journal **2021**, 20, 1778-1793.
49. Pathirage, C.S.N.; J. Li; L. Li, et al. Structural damage identification based on autoencoder neural networks and deep learning. Engineering Structures **2018**, 172, 13-28.
50. Liu, D.; Y.H. Bao; Y.Y. He, and L.K. Zhang. A Data Loss Recovery Technique Using EMD-BiGRU Algorithm for Structural Health Monitoring. Applied Sciences-Basel **2021**, 11, 26.

51. Tsuchimoto, K.; Y. Narazaki; V. Hoskere, and B.F. Spencer. Rapid postearthquake safety evaluation of buildings using sparse acceleration measurements. Structural Health Monitoring-an International Journal **2021**, 20, 1822-1840.
52. Cofre-Martel, S.; P. Kobrich; E.L. Droguett, and V. Meruane. Deep Convolutional Neural Network-Based Structural Damage Localization and Quantification Using Transmissibility Data. Shock and Vibration **2019**, 2019, 27.
53. Merenda, M.; F.G. Pratico; R. Fedele; R. Carotenuto, and F.G. Della Corte. A Real-Time Decision Platform for the Management of Structures and Infrastructures. Electronics **2019**, 8, 22.
54. Mustapha, S.; A. Kassir; K. Hassoun; Z. Dawy, and H. Abi-Rached. Estimation of crowd flow and load on pedestrian bridges using machine learning with sensor fusion. Automation in Construction **2020**, 112, 17.
55. Yang, J.; F. Yang; Y. Zhou, et al. A data-driven structural damage detection framework based on parallel convolutional neural network and bidirectional gated recurrent unit. Information Sciences **2021**, 566, 103-117.
56. Nguyen, D.H.; Q.B. Nguyen; T. Bui-Tien, et al. Damage detection in girder bridges using modal curvatures gapped smoothing method and Convolutional Neural Network: Application to Bo Nghi bridge. **2020**, 109, 102728.
57. Yang, J.; L. Zhang; C. Chen, et al. A hierarchical deep convolutional neural network and gated recurrent unit framework for structural damage detection. Information Sciences **2020**, 540, 117-130.
58. Jian, X.; H. Zhong; Y. Xia, and L. Sun. Faulty data detection and classification for bridge structural health monitoring via statistical and deep-learning approach. Structural Control & Health Monitoring **2021**, 28,
59. Ni, F.; J. Zhang, and M.N. Noori. Deep learning for data anomaly detection and data compression of a long-span suspension bridge. Computer-Aided Civil and Infrastructure Engineering **2020**, 35, 685-700.
60. Pham-Bao, T.; N. Ngo-Kieu; L. Vuong-Cong, and T. Nguyen-Nhat. Energy dissipation-based material deterioration assessment using random decrement technique and convolutional neural network: A case study of Saigon bridge in Ho Chi Minh City, Vietnam. Structural Control & Health Monitoring **2022**, 29, 24.
61. Ma, X.; Y. Lin; Z. Nie, and H. Ma. Structural damage identification based on unsupervised feature-extraction via Variational Auto-encoder. Measurement **2020**, 160,
62. Fan, G.; J. Li; H. Hao, and Y. Xin. Data driven structural dynamic response reconstruction using segment based generative adversarial networks. Engineering Structures **2021**, 234,
63. Zhou, Y.; Y. Pei; S. Zhou, et al. Novel methodology for identifying the weight of moving vehicles on bridges using structural response pattern extraction and deep learning algorithms. Measurement **2021**, 168,
64. Sajedi, S. and X. Liang. Deep generative Bayesian optimization for sensor placement in structural health monitoring. Computer-Aided Civil and Infrastructure Engineering **2022**, 37, 1109-1127.
65. Li, L.; G. Liu; L. Zhang, and Q. Li. FS-LSTM-Based Sensor Fault and Structural Damage Isolation in SHM. Ieee Sensors Journal **2021**, 21, 3250-3259.
66. Mantawy, I.M.; M.O.J.S.C. Mantawy, and H. Monitoring. Convolutional neural network based structural health monitoring for rocking bridge system by encoding time-series into images. **2022**, 29, e2897.

67. Khodabandehlou, H.; G. Pekcan, and M.S. Fadali. Vibration-based structural condition assessment using convolution neural networks. Structural Control & Health Monitoring **2019**, 26,
68. Kanarachos, S.; S.-R.G. Christopoulos; A. Chroneos, and M.E. Fitzpatrick. Detecting anomalies in time series data via a deep learning algorithm combining wavelets, neural networks and Hilbert transform. Expert Systems with Applications **2017**, 85, 292-304.
69. Wang, L.; J. Dang; X. Wang, and A. Shrestha. Waveform-based fracture identification of steel beam ends using convolutional neural networks. Structural Control & Health Monitoring **2021**, 28,
70. Dang, H.V.; H. Tran-Ngoc; T.V. Nguyen, et al. Data-Driven Structural Health Monitoring Using Feature Fusion and Hybrid Deep Learning. Ieee Transactions on Automation Science and Engineering **2021**, 18, 2087-2103.
71. Abdeljaber, O.; O. Avci; M.S. Kiranyaz, et al. 1-D CNNs for structural damage detection: Verification on a structural health monitoring benchmark data. Neurocomputing **2018**, 275, 1308-1317.
72. Oh, B.K. and J. Kim. Multi-Objective Optimization Method to Search for the Optimal Convolutional Neural Network Architecture for Long-Term Structural Health Monitoring. Ieee Access **2021**, 9, 44738-44750.
73. Lei, Y.; Y.X. Zhang; J.N. Mi; W.F. Liu, and L.J. Liu. Detecting structural damage under unknown seismic excitation by deep convolutional neural network with wavelet-based transmissibility data. Structural Health Monitoring-an International Journal **2021**, 20, 1583-1596.
74. Bao, X.X.; T.X. Fan; C. Shi, and G.L. Yang. One-dimensional convolutional neural network for damage detection of jacket-type offshore platforms. Ocean Engineering **2021**, 219, 20.
75. Zhang, R.Y.; L.B. Meng; Z. Mao, and H. Sun. Spatiotemporal Deep Learning for Bridge Response Forecasting. Journal of Structural Engineering **2021**, 147, 9.
76. Zhao, H.W.; Y.L. Ding; A.Q. Li; Z.Z. Ren, and K. Yang. Live-load strain evaluation of the prestressed concrete box-girder bridge using deep learning and clustering. Structural Health Monitoring-an International Journal **2020**, 19, 1051-1063.
77. Song, Q.S.; Y. Chen; E.A. Oskoui, et al. Micro-crack detection method of steel beam surface using stacked autoencoders on massive full-scale sensing strains. Structural Health Monitoring-an International Journal **2020**, 19, 1175-1187.
78. Pal, J.; S. Sikdar, and S. Banerjee. A deep-learning approach for health monitoring of a steel frame structure with bolted connections. Structural Control & Health Monitoring **2022**, 29,
79. Oh, B.K. and J. Kim. Optimal architecture of a convolutional neural network to estimate structural responses for safety evaluation of the structures. Measurement **2021**, 177,
80. Li, Y.; P. Ni; L. Sun, and W. Zhu. A convolutional neural network-based full-field response reconstruction framework with multitype inputs and outputs. Structural Control & Health Monitoring **2022**, 29,
81. Zhao, H.; Y. Ding; A. Li; W. Sheng, and F. Geng. Digital modeling on the nonlinear mapping between multi-source monitoring data of in-service bridges. Structural Control & Health Monitoring **2020**, 27,
82. Oh, B.K.; B. Glisic; Y. Kim, and H.S. Park. Convolutional neural network-based wind-induced response estimation model for tall buildings. Computer-Aided Civil and Infrastructure Engineering **2019**, 34, 843-858.

83. Oh, B.K.; H.S. Park, and B. Glisic. Prediction of long-term strain in concrete structure using convolutional neural networks, air temperature and time stamp of measurements. Automation in Construction **2021**, 126,
84. Jiang, H.; C. Wan; K. Yang; Y. Ding, and S. Xue. Continuous missing data imputation with incomplete dataset by generative adversarial networks-based unsupervised learning for long-term bridge health monitoring. Structural Health Monitoring-an International Journal **2022**, 21, 1093-1109.
85. Song, Q.S.; C. Zhang; G.W. Tang, and F. Ansari. Deep learning method for detection of structural microcracks by brillouin scattering based distributed optical fiber sensors. Smart Materials and Structures **2020**, 29, 12.
86. Deng, Y.; H.W. Ju; Y.H. Li; Y.A. Hu, and A.Q. Li. Abnormal Data Recovery of Structural Health Monitoring for Ancient City Wall Using Deep Learning Neural Network. International Journal of Architectural Heritage **2022**, 19.
87. Oh, B.K.; H.S. Park, and B. Glisic. Time-dependent structural response estimation method for concrete structures using time information and convolutional neural networks. Engineering Structures **2023**, 275, 12.
88. Cristiani, D.; F. Falcetelli; N. Yue, et al. Strain-based delamination prediction in fatigue loaded CFRP coupon specimens by deep learning and static loading data. Composites Part B-Engineering **2022**, 241, 12.
89. Hou, J.L.; H.C. Jiang; C.F. Wan, et al. Deep learning and data augmentation based data imputation for structural health monitoring system in multi-sensor damaged state. Measurement **2022**, 196, 17.
90. Tan, X.Y.; W.Z. Chen; X.J. Tan; T. Zou, and B.W. Du. Prediction for the future mechanical behavior of underwater shield tunnel fusing deep learning algorithm on SHM data. Tunnelling and Underground Space Technology **2022**, 125, 10.
91. Li, Z.Q.; D.S. Li, and T.S. Sun. A Transformer-Based Bridge Structural Response Prediction Framework. Sensors **2022**, 22, 14.
92. Oh, B.K.; B. Glisic; Y. Kim, and H.S. Park. Convolutional neural network-based data recovery method for structural health monitoring. Structural Health Monitoring-an International Journal **2020**, 19, 1821-1838.
93. Yue, Z.X.; Y.L. Ding; H.W. Zhao, and Z.W. Wang. Case Study of Deep Learning Model of Temperature-Induced Deflection of a Cable-Stayed Bridge Driven by Data Knowledge. Symmetry-Basel **2021**, 13, 13.
94. Qu, X.D.; J. Yang, and M. Chang. A Deep Learning Model for Concrete Dam Deformation Prediction Based on RS-LSTM. Journal of Sensors **2019**, 2019, 14.
95. Oh, B.K.; W.C. Jung, and H.S. Park. Artificial intelligence-based damage localization method for building structures using correlation of measured structural responses. Engineering Applications of Artificial Intelligence **2023**, 121, 17.
96. Oh, B.K. and H.S. Park. Urban safety network for long-term structural health monitoring of buildings using convolutional neural network. Automation in Construction **2022**, 137, 14.
97. Lee, K.H.; N. Byun, and D. Shin. A Damage Localization Approach for Rahmen Bridge Based on Convolutional Neural Network. Ksce Journal of Civil Engineering **2020**, 24, 1-9.
98. Chen, Y.L.; Y. Chiang; P.H. Chiu, et al. High-Dimensional Phase Space Reconstruction with a Convolutional Neural Network for Structural Health Monitoring. Sensors **2021**, 21, 15.

99. Dang, H.V.; M. Raza; T.V. Nguyen; T. Bui-Tien, and H.X. Nguyen. Deep learning-based detection of structural damage using time-series data. Structure and Infrastructure Engineering **2021**, 17, 1474-1493.
100. Guo, T.; L.P. Wu; C.J. Wang, and Z.L. Xu. Damage detection in a novel deep-learning framework: a robust method for feature extraction. Structural Health Monitoring-an International Journal **2020**, 19, 424-442.
101. Liu, H. and Y.F. Zhang. Image-driven structural steel damage condition assessment method using deep learning algorithm. Measurement **2019**, 133, 168-181.
102. Won, J.; J.W. Park; S. Jang; K. Jin, and Y. Kim. Automated Structural Damage Identification Using Data Normalization and 1-Dimensional Convolutional Neural Network. Applied Sciences-Basel **2021**, 11, 14.
103. Li, M.Y.; D.W. Jia; Z.Y. Wu; S.M. Qiu, and W. He. Structural damage identification using strain mode differences by the iFEM based on the convolutional neural network (CNN). Mechanical Systems and Signal Processing **2022**, 165, 21.
104. Rosafalco, L.; M. Torzoni; A. Manzoni; S. Mariani, and A. Corigliano. Online structural health monitoring by model order reduction and deep learning algorithms. Computers & Structures **2021**, 255, 18.
105. Oh, B.K.; S.H. Lee, and H.S. Park. Damage localization method for building structures based on the interrelation of dynamic displacement measurements using convolutional neural network. Structural Control & Health Monitoring **2020**, 27, 16.
106. Li, Z.Y.; P. Xu; J. Xing, and C.X. Yang. SDFormer: A Novel Transformer Neural Network for Structural Damage Identification by Segmenting the Strain Field Map. Sensors **2022**, 22, 19.
107. Almutairi, M.; N. Nikitas; O. Abdeljaber; O. Avci, and M. Bocian. A methodological approach towards evaluating structural damage severity using 1D CNNs. Structures **2021**, 34, 4435-4446.
108. Gulgec, N.S.; M. Takac, and S.N. Pakzad. Uncertainty quantification in digital image correlation for experimental evaluation of deep learning based damage diagnostic. Structure and Infrastructure Engineering **2021**, 17, 1459-1473.
109. Gulgec, N.S.; M. Takac, and S.N. Pakzad. Convolutional Neural Network Approach for Robust Structural Damage Detection and Localization. Journal of Computing in Civil Engineering **2019**, 33, 11.
110. Huang, Y. and S.Y. Huang. Neural network-based prediction of topside mass of an in-service jacket platform. Ocean Engineering **2022**, 246, 12.
111. Abbas, N.; T. Umar; R. Salih, et al. Structural Health Monitoring of Underground Metro Tunnel by Identifying Damage Using ANN Deep Learning Auto-Encoder. Applied Sciences-Basel **2023**, 13, 19.
112. Sarwar, M.Z. and D. Cantero. Vehicle assisted bridge damage assessment using probabilistic deep learning. Measurement **2023**, 206, 17.
113. Sajedi, S. and X. Liang. Trident: A Deep Learning Framework for High-Resolution Bridge Vibration Monitoring. Applied Sciences-Basel **2022**, 12, 13.
114. Wang, L.X.; H.B. Liu; Z.H. Chen; F. Zhang, and L.L. Guo. Combined digital twin and hierarchical deep learning approach for intelligent damage identification in cable dome structure. Engineering Structures **2023**, 274, 18.

115. Toan, P.B.; N.N. Tam, and N.K. Nhi. A novel approach to investigate the mechanical properties of the material for bridge health monitoring using convolutional neural network. Structure and Infrastructure Engineering **2022**, 21.
116. Xu, Z.F.; M. Bashir; Y. Yang, et al. Multisensory collaborative damage diagnosis of a 10 MW floating offshore wind turbine tendons using multi-scale convolutional neural network with attention mechanism. Renewable Energy **2022**, 199, 21-34.
117. Alcantara, E.A.M. and T. Saito. Convolutional Neural Network-Based Rapid Post-Earthquake Structural Damage Detection: Case Study. Sensors **2022**, 22, 22.
118. Teng, S.; X.D. Chen; G.F. Chen; L. Cheng, and D. Bassir. Structural damage detection based on convolutional neural networks and population of bridges. Measurement **2022**, 202, 12.
119. Parziale, M.; L. Lomazzi; M. Giglio, and F. Cadini. Vibration-based structural health monitoring exploiting a combination of convolutional neural networks and autoencoders for temperature effects neutralization. Structural Control & Health Monitoring **2022**, 29, 20.
120. Wang, Y.S.; Q.T. Luo; H. Xie; Q. Li, and G.Y. Sun. Digital image correlation (DIC) based damage detection for CFRP laminates by using machine learning based image semantic segmentation. International Journal of Mechanical Sciences **2022**, 230, 13.
121. Liu, B.; Q. Xu; J.Y. Chen; J. Li, and M.M. Wang. A New Framework for Isolating Sensor Failures and Structural Damage in Noisy Environments Based on Stacked Gated Recurrent Unit Neural Networks. Buildings **2022**, 12, 23.
122. Torzoni, M.; A. Manzoni, and S. Mariani. Structural health monitoring of civil structures: A diagnostic framework powered by deep metric learning. Computers & Structures **2022**, 271, 21.
123. Ni, P.; L.M. Sun; J.P. Yang, and Y.X. Li. Multi-End Physics-Informed Deep Learning for Seismic Response Estimation. Sensors **2022**, 22, 23.
124. Parisi, F.; A.M. Mangini; M.P. Fanti, and J.M. Adam. Automated location of steel truss bridge damage using machine learning and raw strain sensor data. Automation in Construction **2022**, 138, 13.
125. Min, S.; K. Jeong; Y. Noh; D. Won, and S. Kim. Damage detection for tethers of submerged floating tunnels based on convolutional neural networks. Ocean Engineering **2022**, 250, 18.
126. Dang, V.H.; T.C. Vu; B.D. Nguyen; Q.H. Nguyen, and T.D. Nguyen. Structural damage detection framework based on graph convolutional network directly using vibration data. Structures **2022**, 38, 40-51.
127. Pathirage, C.S.N.; J. Li; L. Li; H. Hao, and W.Q. Liu. Application of deep autoencoder model for structural condition monitoring. Journal of Systems Engineering and Electronics **2018**, 29, 873-880.
128. Ibrahim, A.; A. Eltawil; Y.S. Na, and S. El-Tawil. A Machine Learning Approach for Structural Health Monitoring Using Noisy Data Sets. Ieee Transactions on Automation Science and Engineering **2020**, 17, 900-908.
129. Lei, X.M.; L.M. Sun, and Y. Xia. Lost data reconstruction for structural health monitoring using deep convolutional generative adversarial networks. Structural Health Monitoring-an International Journal **2021**, 20, 2069-2087.
130. Wu, Y.H.; L. Deng, and W. He. BwimNet: A Novel Method for Identifying Moving Vehicles Utilizing a Modified Encoder-Decoder Architecture. Sensors **2020**, 20, 23.
131. Chen, M.; W.M. Zhai; S.Y. Zhu; L. Xu, and Y. Sun. Vibration-based damage detection of rail fastener using fully convolutional networks. Vehicle System Dynamics **2022**, 60, 2191-2210.

132. Liu, H. and Y. Zhang. Deep learning-based brace damage detection for concentrically braced frame structures under seismic loadings. Advances in Structural Engineering **2019**, 22, 3473-3486.
133. Sajedi, S.O. and X. Liang. Vibration-based semantic damage segmentation for large-scale structural health monitoring. Computer-Aided Civil and Infrastructure Engineering **2020**, 35, 579-596.
134. Rastin, Z.; G.G. Amiri, and E. Darvishan. Generative Adversarial Network for Damage Identification in Civil Structures. Shock and Vibration **2021**, 2021, 12.
135. Silva, M.; A. Santos; R. Santos, et al. Deep principal component analysis: An enhanced approach for structural damage identification. Structural Health Monitoring-an International Journal **2019**, 18, 1444-1463.
136. Uwanuakwa, I.D. Deep Learning Modelling and Generalisation of Carbonation Depth in Fly Ash Blended Concrete. Arabian Journal for Science and Engineering **2021**, 46, 4731-4746.
137. Ji, Z.W.; J.H. Gong, and J.R. Feng. A Novel Deep Learning Approach for Anomaly Detection of Time Series Data. Scientific Programming **2021**, 2021, 11.
138. Zhang, Y. and Y. Lei. Data Anomaly Detection of Bridge Structures Using Convolutional Neural Network Based on Structural Vibration Signals. Symmetry-Basel **2021**, 13,
139. Sajedi, S. and X. Liang. Dual Bayesian inference for risk-informed vibration-based damage diagnosis. Computer-Aided Civil and Infrastructure Engineering **2021**, 36, 1168-1184.
140. Azimi, M. and G. Pekcan. Structural health monitoring using extremely compressed data through deep learning. Computer-Aided Civil and Infrastructure Engineering **2020**, 35, 597-614.
141. Wang, X.W.; X.N. Zhang, and M.M. Shahzad. A novel structural damage identification scheme based on deep learning framework. Structures **2021**, 29, 1537-1549.
142. He, Y.Y.; H.Y. Chen; D. Liu, and L.K. Zhang. A Framework of Structural Damage Detection for Civil Structures Using Fast Fourier Transform and Deep Convolutional Neural Networks. Applied Sciences-Basel **2021**, 11, 22.
143. Liu, T.W.; H. Xu; M. Ragulskis; M.S. Cao, and W. Ostachowicz. A Data-Driven Damage Identification Framework Based on Transmissibility Function Datasets and One-Dimensional Convolutional Neural Networks: Verification on a Structural Health Monitoring Benchmark Structure. Sensors **2020**, 20, 25.
144. Avci, O.; O. Abdeljaber; S. Kiranyaz; M. Hussein, and D.J. Inman. Wireless and real-time structural damage detection: A novel decentralized method for wireless sensor networks. Journal of Sound and Vibration **2018**, 424, 158-172.
145. Fathnejat, H.; B. Ahmadi-Nedushan; S. Hosseininejad; M. Noori, and W.A. Altabey. A data-driven structural damage identification approach using deep convolutional-attention-recurrent neural architecture under temperature variations. Engineering Structures **2023**, 276, 14.
146. Xue, Y.F.; C.Z. Cai, and Y.L. Chi. Frame Structure Fault Diagnosis Based on a High-Precision Convolution Neural Network. Sensors **2022**, 22, 16.
147. Chamangard, M.; G.G. Amiri; E. Darvishan, and Z. Rastin. Transfer Learning for CNN-Based Damage Detection in Civil Structures with Insufficient Data. Shock and Vibration **2022**, 2022, 14.
148. Liao, S.Y.; H.J. Liu; J.X. Yang, and Y.X. Ge. A channel-spatial-temporal attention-based network for vibration-based damage detection. Information Sciences **2022**, 606, 213-229.

149. Jiang, K.J.; Q. Han, and X.L. Du. Lost data neural semantic recovery framework for structural health monitoring based on deep learning. Computer-Aided Civil and Infrastructure Engineering **2022**, 37, 1160-1187.
150. Li, S.J.; F.Y. Liu; G.L. Peng, et al. A Lightweight SHM Framework Based on Adaptive Multisensor Fusion Network and Multigeneration Knowledge Distillation. Ieee Transactions on Instrumentation and Measurement **2022**, 71, 19.
151. Sony, S.; S. Gamage; A. Sadhu, and J. Samarabandu. Vibration-based multiclass damage detection and localization using long short-term memory networks. Structures **2022**, 35, 436-451.
152. Soleimani-Babakamali, M.H.; R. Sepasdar; K. Nasrollahzadeh; I. Lourentzou, and R. Sarlo. Toward a general unsupervised novelty detection framework in structural health monitoring. Computer-Aided Civil and Infrastructure Engineering **2022**, 37, 1128-1145.
153. Sony, S.; S. Gamage; A. Sadhu, and J. Samarabandu. Multiclass Damage Identification in a Full-Scale Bridge Using Optimally Tuned One-Dimensional Convolutional Neural Network. Journal of Computing in Civil Engineering **2022**, 36, 14.
154. Rastin, Z.; G. Ghodrati Amiri, and E. Darvishan. Unsupervised Structural Damage Detection Technique Based on a Deep Convolutional Autoencoder. Shock and Vibration **2021**, 2021,
155. Li, S.; L. Jin; Y. Qiu; M.M. Zhang, and J. Wang. Signal Anomaly Detection of Bridge SHM System Based on Two-Stage Deep Convolutional Neural Networks. Structural Engineering International **2023**, 33, 74-83.
156. Fan, G.; J. Li, and H. Hao. Vibration signal denoising for structural health monitoring by residual convolutional neural networks. Measurement **2020**, 157, 15.
157. Tang, Z.; Z. Chen; Y. Bao, and H. Li. Convolutional neural network-based data anomaly detection method using multiple information for structural health monitoring. Structural Control & Health Monitoring **2019**, 26,
158. Zhang, H.; J. Lin; J.D. Hua; F. Gao, and T. Tong. Data Anomaly Detection for Bridge SHM Based on CNN Combined with Statistic Features. Journal of Nondestructive Evaluation **2022**, 41, 13.
159. Li, Y.; T. Bao; Z. Gao, et al. A new dam structural response estimation paradigm powered by deep learning and transfer learning techniques. Structural Health Monitoring-an International Journal **2022**, 21, 770-787.
160. Li, Y.; T. Bao; H. Chen, et al. A large-scale sensor missing data imputation framework for dams using deep learning and transfer learning strategy. Measurement **2021**, 178,
161. Xiong, Q.S.; H.B. Xiong; C. Yuan, and Q.Z. Kong. A novel deep convolutional image-denoiser network for structural vibration signal denoising. Engineering Applications of Artificial Intelligence **2023**, 117, 13.
162. Li, Y.; T. Bao; J. Gong; X. Shu, and K. Zhang. The Prediction of Dam Displacement Time Series Using STL, Extra-Trees, and Stacked LSTM Neural Network. Ieee Access **2020**, 8, 94440-94452.
163. Deng, L.; H.H. Chu; P. Shi; W. Wang, and X. Kong. Region-Based CNN Method with Deformable Modules for Visually Classifying Concrete Cracks. Applied Sciences-Basel **2020**, 10, 18.
164. Pham, H.C.; Q.B. Ta; J.T. Kim, et al. Bolt-Loosening Monitoring Framework Using an Image-Based Deep Learning and Graphical Model. Sensors **2020**, 20, 19.
165. Andrushia, A.D.; N. Anand; E. Lubloy, and G.P. Arulraj. Deep learning based thermal crack detection on structural concrete exposed to elevated temperature. Advances in Structural Engineering **2021**, 24, 1896-1909.

166. Huang, H.W.; Q.T. Li, and D.M. Zhang. Deep learning based image recognition for crack and leakage defects of metro shield tunnel. Tunnelling and Underground Space Technology **2018**, 77, 166-176.
167. Wang, S.; C. Liu, and Y.H. Zhang. Fully convolution network architecture for steel-beam crack detection in fast-stitching images. Mechanical Systems and Signal Processing **2022**, 165, 20.
168. Hallee, M.J.; R.K. Napolitano; W.F. Reinhart, and B. Glisic. Crack Detection in Images of Masonry Using CNNs. Sensors **2021**, 21, 19.
169. Dong, C.Z.; O. Celik; F.N. Catbas; E.J. O'Brien, and S. Taylor. Structural displacement monitoring using deep learning-based full field optical flow methods. Structure and Infrastructure Engineering **2020**, 16, 51-71.
170. Xu, X.Y. and H. Yang. Vision Measurement of Tunnel Structures with Robust Modelling and Deep Learning Algorithms. Sensors **2020**, 20, 15.
171. Yang, H.; H.C. Xu; S.J. Jiao, and F.D. Yin. Semantic Image Segmentation Based Cable Vibration Frequency Visual Monitoring Using Modified Convolutional Neural Network with Pixel-wise Weighting Strategy. Remote Sensing **2021**, 13, 29.
172. Perez, H.; J.H.M. Tah, and A. Mosavi. Deep Learning for Detecting Building Defects Using Convolutional Neural Networks. Sensors **2019**, 19, 22.
173. Wei, W.; L.Y. Ding; H.B. Luo; C. Li, and G.W. Li. Automated bughole detection and quality performance assessment of concrete using image processing and deep convolutional neural networks. Construction and Building Materials **2021**, 281, 11.
174. Kulkarni, N.N.; K. Raisi; N.A. Valente, et al. Deep learning augmented infrared thermography for unmanned aerial vehicles structural health monitoring of roadways. Automation in Construction **2023**, 148, 13.
175. Sun, C.J.; D.L. Gu, and X.Z. Lu. Three-dimensional structural displacement measurement using monocular vision and deep learning based pose estimation. Mechanical Systems and Signal Processing **2023**, 190, 26.
176. Ali, L.; H.A. Jassmi; W. Khan, and F. Alnajjar. Crack45K: Integration of Vision Transformer with Tubularity Flow Field (TuFF) and Sliding-Window Approach for Crack-Segmentation in Pavement Structures. Buildings **2023**, 13, 19.
177. Wang, L.L.; J.J. Li, and F. Kang. Crack Location and Degree Detection Method Based on YOLOX Model. Applied Sciences-Basel **2022**, 12, 17.
178. Qiu, D.W.; H.R. Liang; Z.L. Wang; Y.C. Tong, and S.S. Wan. Hybrid-Supervised-Learning-Based Automatic Image Segmentation for Water Leakage in Subway Tunnels. Applied Sciences-Basel **2022**, 12, 20.
179. Qi, Y.Z.; C. Yuan; P.Z. Li, and Q.Z. Kong. Damage analysis and quantification of RC beams assisted by Damage-T Generative Adversarial Network. Engineering Applications of Artificial Intelligence **2023**, 117, 11.
180. Zhao, S.Z.; F. Kang, and J.J. Li. Non-Contact Crack Visual Measurement System Combining Improved U-Net Algorithm and Canny Edge Detection Method with Laser Rangefinder and Camera. Applied Sciences-Basel **2022**, 12, 30.
181. Li, Z.J.; K. Adamu; K. Yan, et al. Detection of Nut-Bolt Loss in Steel Bridges Using Deep Learning Techniques. Sustainability **2022**, 14, 18.

182. Lydon, D.; R. Kromanis; M. Lydon; J. Early, and S. Taylor. Use of a roving computer vision system to compare anomaly detection techniques for health monitoring of bridges. Journal of Civil Structural Health Monitoring **2022**, 12, 1299-1316.
183. Li, Z.Q. and D.S. Li. A high-frequency feature enhancement network for the surface defect detection of welded rebar. Structural Control & Health Monitoring **2022**, 29, 13.
184. Song, Q.S.; J.R. Wu; H.L. Wang; Y.S. An, and G.W. Tang. Computer vision-based illumination-robust and multi-point simultaneous structural displacement measuring method. Mechanical Systems and Signal Processing **2022**, 170, 14.
185. Kong, S.Y.; J.S. Fan; Y.F. Liu; X.C. Wei, and X.W. Ma. Automated crack assessment and quantitative growth monitoring. Computer-Aided Civil and Infrastructure Engineering **2021**, 36, 656-674.
186. Ma, Z.R. and L. Gao. Predicting Mechanical State of High-Speed Railway Elevated Station Track System Using a Hybrid Prediction Model. Ksce Journal of Civil Engineering **2021**, 25, 2474-2486.
187. Shao, Y.D.; L. Li; J. Li; S.J. An, and H. Hao. Computer vision based target-free 3D vibration displacement measurement of structures. Engineering Structures **2021**, 246, 16.
188. Deng, G.J.; Z.X. Zhou; X. Chu, and S. Shao. Identification of Behavioral Features of Bridge Structure Based on Static Image Sequences. Advances in Civil Engineering **2020**, 2020, 16.
189. Yuan, C.; B. Xiong; X.Q. Li; X.H. Sang, and Q.Z. Kong. A novel intelligent inspection robot with deep stereo vision for three-dimensional concrete damage detection and quantification. Structural Health Monitoring-an International Journal **2022**, 21, 788-802.
190. Chen, F.C. and M.R. Jahanshahi. ARF-Crack: rotation invariant deep fully convolutional network for pixel-level crack detection. Machine Vision and Applications **2020**, 31, 12.
191. Attard, L.; C.J. Debono; G. Valentino, and M. Di Castro. Vision-Based Tunnel Lining Health Monitoring via Bi-Temporal Image Comparison and Decision-Level Fusion of Change Maps. Sensors **2021**, 21, 20.
192. Elhariri, E.; N. El-Bendary, and S.A. Taie. Using Hybrid Filter-Wrapper Feature Selection With Multi-Objective Improved-Salp Optimization for Crack Severity Recognition. Ieee Access **2020**, 8, 84290-84315.
193. Ye, X.W.; T. Jin; Z.X. Li, et al. Structural Crack Detection from Benchmark Data Sets Using Pruned Fully Convolutional Networks. Journal of Structural Engineering **2021**, 147, 13.
194. Zhang, L.Y.; G.C. Zhou; Y. Han; H.L. Lin, and Y.Y. Wu. Application of Internet of Things Technology and Convolutional Neural Network Model in Bridge Crack Detection. Ieee Access **2018**, 6, 39442-39451.
195. Dorafshan, S.; R.J. Thomas, and M. Maguire. Comparison of deep convolutional neural networks and edge detectors for image-based crack detection in concrete. Construction and Building Materials **2018**, 186, 1031-1045.
196. Ren, Y.; J. Huang; Z. Hong, et al. Image-based concrete crack detection in tunnels using deep fully convolutional networks. Construction and Building Materials **2020**, 234,
197. Kumar, P.; A. Sharma, and S.R. Kota. Automatic Multiclass Instance Segmentation of Concrete Damage Using Deep Learning Model. Ieee Access **2021**, 9, 90330-90345.
198. Li, C.; P. Xu; L. Niu, et al. Tunnel crack detection using coarse-to-fine region localization and edge detection. Wiley Interdisciplinary Reviews-Data Mining and Knowledge Discovery **2019**, 9,

199. Chou, J.-S.; M.A. Karundeng; D.-N. Truong, and M.-Y. Cheng. Identifying deflections of reinforced concrete beams under seismic loads by bio-inspired optimization of deep residual learning. Structural Control & Health Monitoring **2022**, 29,
200. Feng, C.C.; H. Zhang; S. Wang, et al. Structural Damage Detection using Deep Convolutional Neural Network and Transfer Learning. Ksce Journal of Civil Engineering **2019**, 23, 4493-4502.
201. Zhang, Y.; X.W. Sun; K.J. Loh, et al. Autonomous bolt loosening detection using deep learning. Structural Health Monitoring-an International Journal **2020**, 19, 105-122.
202. Zhang, Y.; P. Liu, and X.F. Zhao. Structural displacement monitoring based on mask regions with convolutional neural network. Construction and Building Materials **2021**, 267, 8.
203. Zhao, X.F.; Y. Zhang, and N.N. Wang. Bolt loosening angle detection technology using deep learning. Structural Control & Health Monitoring **2019**, 26, 14.
204. Zhang, Y.; X.F. Zhao, and P. Liu. Multi-Point Displacement Monitoring Based on Full Convolutional Neural Network and Smartphone. Ieee Access **2019**, 7, 139628-139634.
205. Yao, G.; Y.J. Sun; M.P. Wong, and X.N. Lv. A Real-Time Detection Method for Concrete Surface Cracks Based on Improved YOLOv4. Symmetry-Basel **2021**, 13, 16.
206. Roberts, R.; G. Giancontieri; L. Inzerillo, and G. Di Mino. Towards Low-Cost Pavement Condition Health Monitoring and Analysis Using Deep Learning. Applied Sciences-Basel **2020**, 10, 22.
207. Kao, S.P.; Y.C. Chang, and F.L. Wang. Combining the YOLOv4 Deep Learning Model with UAV Imagery Processing Technology in the Extraction and Quantization of Cracks in Bridges. Sensors **2023**, 23, 17.
208. Pan, X.; T.Y. Yang; Y.F. Xiao; H.C. Yao, and H. Adeli. Vision-based real-time structural vibration measurement through deep-learning-based detection and tracking methods. Engineering Structures **2023**, 281, 12.
209. Lu, Q.Z.; Y.C. Jing, and X.F. Zhao. Bolt Loosening Detection Using Key-Point Detection Enhanced by Synthetic Datasets. Applied Sciences-Basel **2023**, 13, 21.
210. Kang, D.H. and Y.J. Cha. Autonomous UAVs for Structural Health Monitoring Using Deep Learning and an Ultrasonic Beacon System with Geo-Tagging. Computer-Aided Civil and Infrastructure Engineering **2018**, 33, 885-902.
211. Saleem, M.R.; J.W. Park; J.H. Lee; H.J. Jung, and M.Z. Sarwar. Instant bridge visual inspection using an unmanned aerial vehicle by image capturing and geo-tagging system and deep convolutional neural network. Structural Health Monitoring-an International Journal **2021**, 20, 1760-1777.
212. Zhang, C.; Y. Tian, and J. Zhang. Complex image background segmentation for cable force estimation of urban bridges with drone-captured video and deep learning. Structural Control & Health Monitoring **2022**, 29,
213. Geetha, G.K.; H.J. Yang, and S.H. Sim. Fast Detection of Missing Thin Propagating Cracks during Deep-Learning-Based Concrete Crack/Non-Crack Classification. Sensors **2023**, 23, 21.
214. Zhao, S.Z.; F. Kang, and J.J. Li. Concrete dam damage detection and localisation based on YOLOv5s-HSC and photogrammetric 3D reconstruction. Automation in Construction **2022**, 143, 18.
215. Agyemang, I.O.; X.L. Zhang; D. Acheampong, et al. Autonomous health assessment of civil infrastructure using deep learning and smart devices. Automation in Construction **2022**, 141, 15.

216. Hou, R.; S. Jeong; J.P. Lynch, and K.H. Law. Cyber-physical system architecture for automating the mapping of truck loads to bridge behavior using computer vision in connected highway corridors. Transportation Research Part C-Emerging Technologies **2020**, 111, 547-571.
217. Jian, X.D.; Y. Xia; J.A. Lozano-Galant, and L.M. Sun. Traffic Sensing Methodology Combining Influence Line Theory and Computer Vision Techniques for Girder Bridges. Journal of Sensors **2019**, 2019, 15.
218. Xia, Y.; X.D. Jian; B. Yan, and D. Su. Infrastructure Safety Oriented Traffic Load Monitoring Using Multi-Sensor and Single Camera for Short and Medium Span Bridges. Remote Sensing **2019**, 11, 21.
219. Zhou, Y.; Y. Pei; Z. Li, et al. Vehicle weight identification system for spatiotemporal load distribution on bridges based on non-contact machine vision technology and deep learning algorithms. Measurement **2020**, 159,
220. Ge, L.; D. Dan, and H. Li. An accurate and robust monitoring method of full-bridge traffic load distribution based on YOLO-v3 machine vision. Structural Control & Health Monitoring **2020**, 27,
221. Zhang, L.X.; J.K. Shen, and B.J. Zhu. A research on an improved Unet-based concrete crack detection algorithm. Structural Health Monitoring-an International Journal **2021**, 20, 1864-1879.
222. Fu, R.H.; H. Xu; Z.J. Wang, et al. Enhanced Intelligent Identification of Concrete Cracks Using Multi-Layered Image Preprocessing-Aided Convolutional Neural Networks. Sensors **2020**, 20, 24.
223. Alipour, M. and D.K. Harris. A big data analytics strategy for scalable urban infrastructure condition assessment using semi-supervised multi-transform self-training. Journal of Civil Structural Health Monitoring **2020**, 10, 313-332.
224. Kim, B.; N. Yuvaraj; K.R.S. Preethaa, and R.A. Pandian. Surface crack detection using deep learning with shallow CNN architecture for enhanced computation. Neural Computing & Applications **2021**, 33, 9289-9305.
225. Altabey, W.A.; M. Noori; T.Y. Wang, et al. Deep Learning-Based Crack Identification for Steel Pipelines by Extracting Features from 3D Shadow Modeling. Applied Sciences-Basel **2021**, 11, 21.
226. Cui, X.N.; Q.C. Wang; J.P. Dai; Y.J. Xue, and Y. Duan. Intelligent crack detection based on attention mechanism in convolution neural network. Advances in Structural Engineering **2021**, 24, 1859-1868.
227. Rao, A.S.; N. Tuan; M. Palaniswami, and N. Tuan. Vision-based automated crack detection using convolutional neural networks for condition assessment of infrastructure. Structural Health Monitoring-an International Journal **2021**, 20, 2124-2142.
228. Deng, W.L.; Y.L. Mou; T. Kashiwa, et al. Vision based pixel-level bridge structural damage detection using a link ASPP network. Automation in Construction **2020**, 110, 9.
229. Park, S.E.; S.H. Eem, and H. Jeon. Concrete crack detection and quantification using deep learning and structured light. Construction and Building Materials **2020**, 252, 8.
230. Wang, S.H.; S.A. Zargar, and F.G. Yuan. Augmented reality for enhanced visual inspection through knowledge-based deep learning. Structural Health Monitoring-an International Journal **2021**, 20, 426-442.
231. Sajedi, S.O. and X. Liang. Uncertainty-assisted deep vision structural health monitoring. Computer-Aided Civil and Infrastructure Engineering **2021**, 36, 126-142.

232. Iyer, S.; T. Velmurugan; A.H. Gandomi, et al. Structural health monitoring of railway tracks using IoT-based multi-robot system. Neural Computing & Applications **2021**, 33, 5897-5915.
233. Li, Z.; H. Zhu, and M. Huang. A Deep Learning-Based Fine Crack Segmentation Network on Full-Scale Steel Bridge Images With Complicated Backgrounds. Ieee Access **2021**, 9, 114989-114997.
234. Islam, M.M.M. and J.-M. Kim. Vision-Based Autonomous Crack Detection of Concrete Structures Using a Fully Convolutional Encoder-Decoder Network. Sensors **2019**, 19,
235. Rubio, J.J.; T. Kashiwa; T. Laiteerapong, et al. Multi-class structural damage segmentation using fully convolutional networks. Computers in Industry **2019**, 112,
236. Ye, X.-W.; T. Jin, and P.-Y. Chen. Structural crack detection using deep learning-based fully convolutional networks. Advances in Structural Engineering **2019**, 22, 3412-3419.
237. Gao, Y.; P. Zhai, and K.M. Mosalam. Balanced semisupervised generative adversarial network for damage assessment from low-data imbalanced-class regime. Computer-Aided Civil and Infrastructure Engineering **2021**, 36, 1094-1113.
238. Panta, M.; M.T. Hoque; M. Abdelguerfi, and M.C. Flanagin. IterLUNet: Deep Learning Architecture for Pixel-Wise Crack Detection in Levee Systems. Ieee Access **2023**, 11, 12249-12262.
239. Arafin, P.; A. Issa, and A. Billah. Performance Comparison of Multiple Convolutional Neural Networks for Concrete Defects Classification. Sensors **2022**, 22, 18.
240. Rosso, M.M.; G. Marasco; S. Aiello, et al. Convolutional networks and transformers for intelligent road tunnel investigations. Computers & Structures **2023**, 275, 22.
241. Ye, X.W.; S.Y. Ma; Z.X. Liu, et al. Post-earthquake damage recognition and condition assessment of bridges using UAV integrated with deep learning approach. Structural Control & Health Monitoring **2022**, 29, 15.
242. Gao, Y.Q. and K.M. Mosalam. Deep learning visual interpretation of structural damage images. Journal of Building Engineering **2022**, 60, 17.
243. Gao, X.J.; C.S. Huang; S. Teng, and G.F. Chen. A Deep-Convolutional-Neural-Network-Based Semi-Supervised Learning Method for Anomaly Crack Detection. Applied Sciences-Basel **2022**, 12, 20.
244. Dunphy, K.; A. Sadhu, and J.F. Wang. Multiclass damage detection in concrete structures using a transfer learning-based generative adversarial networks. Structural Control & Health Monitoring **2022**, 29, 20.
245. Choi, W. and Y.J. Cha. SDDNet: Real-Time Crack Segmentation. Ieee Transactions on Industrial Electronics **2020**, 67, 8016-8025.
246. Chen, G.F.; S. Teng; M.S. Lin; X.M. Yang, and X.L. Sun. Crack Detection Based on Generative Adversarial Networks and Deep Learning. Ksce Journal of Civil Engineering **2022**, 26, 1803-1816.
247. Dunphy, K.; M.N. Fekri; K. Grolinger, and A. Sadhu. Data Augmentation for Deep-Learning-Based Multiclass Structural Damage Detection Using Limited Information. Sensors **2022**, 22, 29.
248. Jang, K.; N. Kim, and Y.-K. An. Deep learning-based autonomous concrete crack evaluation through hybrid image scanning. Structural Health Monitoring-an International Journal **2019**, 18, 1722-1737.

249. Luan, L.L.; J.W. Zheng; M.L. Wang, et al. Extracting full-field subpixel structural displacements from videos via deep learning. Journal of Sound and Vibration **2021**, 505, 19.
250. Lado-Roige, R.; J. Font-More, and M.A. Perez. Learning-based video motion magnification approach for vibration-based damage detection. Measurement **2023**, 206, 10.
251. Shao, Y.D.; L. Li; J. Li; S.J. An, and H. Hao. Target-free 3D tiny structural vibration measurement based on deep learning and motion magnification. Journal of Sound and Vibration **2022**, 538, 20.
252. Ai, L.; V. Soltangharaei, and P. Ziehl. Evaluation of ASR in concrete using acoustic emission and deep learning. Nuclear Engineering and Design **2021**, 380, 10.
253. Sikdar, S.; D.Z. Liu, and A. Kundu. Acoustic emission data based deep learning approach for classification and detection of damage-sources in a composite panel. Composites Part B-Engineering **2022**, 228, 9.
254. Siracusano, G.; F. Garesci; G. Finocchio, et al. Automatic Crack Classification by Exploiting Statistical Event Descriptors for Deep Learning. Applied Sciences-Basel **2021**, 11, 21.
255. Ebrahimkhanlou, A.; B. Dubuc, and S. Salamone. A generalizable deep learning framework for localizing and characterizing acoustic emission sources in riveted metallic panels. Mechanical Systems and Signal Processing **2019**, 130, 248-272.
256. Haile, M.A.; E. Zhu; C. Hsu, and N. Bradley. Deep machine learning for detection of acoustic wave reflections. Structural Health Monitoring-an International Journal **2020**, 19, 1340-1350.
257. Yang, L. and F.Y. Xu. A Novel Acoustic Emission Sources Localization and Identification Method in Metallic Plates Based on Stacked Denoising Autoencoders. Ieee Access **2020**, 8, 141123-141142.
258. Garrett, J.C.; H.F. Mei, and V. Giurgiutiu. An Artificial Intelligence Approach to Fatigue Crack Length Estimation from Acoustic Emission Waves in Thin Metallic Plates. Applied Sciences-Basel **2022**, 12, 17.
259. Hesser, D.F.; S. Mostafavi; G.K. Kocur, and B. Markert. Identification of acoustic emission sources for structural health monitoring applications based on convolutional neural networks and deep transfer learning. Neurocomputing **2021**, 453, 1-12.
260. Ebrahimkhanlou, A. and S. Salamone Single-sensor acoustic emission source localization in plate-like structures: A deep learning approach. in SPIE Conference on Health Monitoring of Structural and Biological Systems XII, Denver, CO.
261. Chen, S.-X.; L. Zhou; Y.-Q. Ni, and X.-Z. Liu. An acoustic-homologous transfer learning approach for acoustic emission-based rail condition evaluation. Structural Health Monitoring-an International Journal **2021**, 20, 2161-2181.
262. Han, G.; Y.-M. Kim; H. Kim, et al. Auto-detection of acoustic emission signals from cracking of concrete structures using convolutional neural networks: Upscaling from specimen. Expert Systems with Applications **2021**, 186,
263. Guo, S.F.; H. Ding; Y.H. Li, et al. A hierarchical deep convolutional regression framework with sensor network fail-safe adaptation for acoustic-emission-based structural health monitoring. Mechanical Systems and Signal Processing **2022**, 181, 17.
264. Zhang, B.; X.B. Hong, and Y. Liu. Distribution adaptation deep transfer learning method for cross-structure health monitoring using guided waves. Structural Health Monitoring-an International Journal **2022**, 21, 853-871.

265. Zhang, B.; X.B. Hong, and Y. Liu. Deep Convolutional Neural Network Probability Imaging for Plate Structural Health Monitoring Using Guided Waves. Ieee Transactions on Instrumentation and Measurement **2021**, 70, 10.
266. Li, Y.T.; T.F. Bao; J. Gong; X.S. Shu, and K. Zhang. The Prediction of Dam Displacement Time Series Using STL, Extra-Trees, and Stacked LSTM Neural Network. Ieee Access **2020**, 8, 94440-94452.
267. Gao, F. and J.D. Hua. Damage characterization using CNN and SAE of broadband Lamb waves. Ultrasonics **2022**, 119, 10.
268. Pandey, P.; A. Rai, and M. Mitra. Explainable 1-D convolutional neural network for damage detection using Lamb wave. Mechanical Systems and Signal Processing **2022**, 164, 12.
269. Zhou, L.; S.-X. Chen; Y.-Q. Ni, and L. Jiang. Pitch-catch UGW-based multiple damage inference: a heterogeneous graph interpretation. Smart Materials and Structures **2022**, 31,
270. Zhang, S.Y.; C.M. Li, and W.J. Ye. Damage localization in plate-like structures using time-varying feature and one-dimensional convolutional neural network. Mechanical Systems and Signal Processing **2021**, 147, 15.
271. Mariani, S.; Q. Rendu; M. Urbani, and C. Sbarufatti. Causal dilated convolutional neural networks for automatic inspection of ultrasonic signals in non-destructive evaluation and structural health monitoring. Mechanical Systems and Signal Processing **2021**, 157, 22.
272. Azuara, G.; M. Ruiz, and E. Barrera. Damage Localization in Composite Plates Using Wavelet Transform and 2-D Convolutional Neural Networks. Sensors **2021**, 21, 16.
273. Chen, J.; W. Wu; Y. Ren, and S. Yuan. Fatigue Crack Evaluation with the Guided Wave-Convolutional Neural Network Ensemble and Differential Wavelet Spectrogram. Sensors **2022**, 22,
274. Lim, H.J. and H. Sohn. Online Stress Monitoring Technique Based on Lamb-wave Measurements and a Convolutional Neural Network Under Static and Dynamic Loadings. Experimental Mechanics **2020**, 60, 171-179.
275. Liao, Y.L.; X.L. Qing; Y.H. Wang, and F.H. Zhang. Damage localization for composite structure using guided wave signals with Gramian angular field image coding and convolutional neural networks. Composite Structures **2023**, 312, 14.
276. Lomazzi, L.; M. Giglio, and F. Cadini. Towards a deep learning-based unified approach for structural damage detection, localisation and quantification. Engineering Applications of Artificial Intelligence **2023**, 121, 15.
277. Sawant, S.; A. Sethi; S. Banerjee, and S. Tallur. Unsupervised learning framework for temperature compensated damage identification and localization in ultrasonic guided wave SHM with transfer learning. Ultrasonics **2023**, 130, 10.
278. Sattarifar, A. and T. Nestorovi. Damage localization and characterization using one-dimensional convolutional neural network and a sparse network of transducers. Engineering Applications of Artificial Intelligence **2022**, 115, 13.
279. Karmakov, S. and M.H.F. Aliabadi. Deep Learning Approach to Impact Classification in Sensorized Panels Using Self-Attention. Sensors **2022**, 22, 17.
280. Farias, S.V.; O. Saotome; H.F.C. Velho, and E.H. Shiguemori. A Damage Detection Method Using Neural Network Optimized by Multiple Particle Collision Algorithm. Journal of Sensors **2021**, 2021, 14.

281. Ijjeh, A.A.; S. Ullah, and P. Kudela. Full wavefield processing by using FCN for delamination detection. Mechanical Systems and Signal Processing **2021**, 153, 16.
282. Zhang, Z.; F.J. Tang; Q. Cao, et al. Deep Learning-Enriched Stress Level Identification of Pretensioned Rods via Guided Wave Approaches. Buildings **2022**, 12, 19.
283. Lomazzi, L.; S. Fabiano; M. Parziale; M. Giglio, and F. Cadini. On the explainability of convolutional neural networks processing ultrasonic guided waves for damage diagnosis. Mechanical Systems and Signal Processing **2023**, 183, 18.
284. Zhang, Z.; H. Pan; X.Y. Wang, and Z.B. Lin. Deep Learning Empowered Structural Health Monitoring and Damage Diagnostics for Structures with Weldment via Decoding Ultrasonic Guided Wave. Sensors **2022**, 22, 30.
285. Jiang, S.Y.; C. Wan; L.G. Sun, and C.B. Du. Flaw classification and detection in thin-plate structures based on scaled boundary finite element method and deep learning. International Journal for Numerical Methods in Engineering **2022**, 123, 4674-4701.
286. Ijjeh, A.A. and P. Kudela. Deep learning based segmentation using full wavefield processing for delamination identification: A comparative study. Mechanical Systems and Signal Processing **2022**, 168, 16.
287. Ullah, S.; A.A. Ijjeh, and P. Kudela. Deep learning approach for delamination identification using animation of Lamb waves. Engineering Applications of Artificial Intelligence **2023**, 117, 17.
288. Dang, D.Z.; C.C. Lai; Y.Q. Ni, et al. Image Classification-Based Defect Detection of Railway Tracks Using Fiber Bragg Grating Ultrasonic Sensors. Applied Sciences-Basel **2023**, 13, 18.
289. de Oliveira, M.A.; A.V. Monteiro, and J. Vieira. A New Structural Health Monitoring Strategy Based on PZT Sensors and Convolutional Neural Network. Sensors **2018**, 18, 21.
290. Monteiro, A.; M. de Oliveira; R. de Oliveira, and T. da Silva. Embedded application of convolutional neural networks on Raspberry Pi for SHM. Electronics Letters **2018**, 54, 680-681.
291. Alazzawi, O. and D.S. Wang. Damage identification using the PZT impedance signals and residual learning algorithm. Journal of Civil Structural Health Monitoring **2021**, 11, 1225-1238.
292. Alazzawi, O. and D. Wang. Deep convolution neural network for damage identifications based on time-domain PZT impedance technique. Journal of Mechanical Science and Technology **2021**, 35, 1809-1819.
293. Miorelli, R.; C. Fisher; A. Kulakovskyi, et al. Defect sizing in guided wave imaging structural health monitoring using convolutional neural networks. Ndt & E International **2021**, 122,
294. Parida, L.; S. Moharana; V.M. Ferreira; S.K. Giri, and G. Ascensao. A Novel CNN-LSTM Hybrid Model for Prediction of Electro-Mechanical Impedance Signal Based Bond Strength Monitoring. Sensors **2022**, 22, 26.
295. Nguyen, T.T.; T.T.V. Phan; D.D. Ho; A.M.S. Pradhan, and T.C. Huynh. Deep learning-based autonomous damage-sensitive feature extraction for impedance-based prestress monitoring. Engineering Structures **2022**, 259, 18.
296. Ahmed, H.; H.M. La, and K. Tran. Rebar detection and localization for bridge deck inspection and evaluation using deep residual networks. Automation in Construction **2020**, 120, 18.
297. Li, S.L.; J. Niu, and Z.L. Li. Novelty detection of cable-stayed bridges based on cable force correlation exploration using spatiotemporal graph convolutional networks. Structural Health Monitoring-an International Journal **2021**, 20, 2216-2228.
298. Son, H.; V.T. Pham; Y. Jang, and S.E. Kim. Damage Localization and Severity Assessment of a Cable-Stayed Bridge Using a Message Passing Neural Network. Sensors **2021**, 21, 21.

299. Son, H.; Y. Jang; S.-E. Kim; D. Kim, and J.-W. Park. Deep Learning-Based Anomaly Detection to Classify Inaccurate Data and Damaged Condition of a Cable-Stayed Bridge. Ieee Access **2021**, 9, 124549-124559.
300. Huang, B.; F. Kang; J.J. Li, and F. Wang. Displacement prediction model for high arch dams using long short-term memory based encoder-decoder with dual-stage attention considering measured dam temperature. Engineering Structures **2023**, 280, 15.
301. Deng, Y.; H.W. Ju; W.Q. Zhai; A.Q. Li, and Y.L. Ding. Correlation model of deflection, vehicle load, and temperature for in-service bridge using deep learning and structural health monitoring. Structural Control & Health Monitoring **2022**, 29, 20.
302. Yue, Z.X.; Y.L. Ding; H.W. Zhao, and Z.W. Wang. Ultra-high precise Stack-LSTM-CNN model of temperature-induced deflection of a cable-stayed bridge for detecting bridge state driven by monitoring data. Structures **2022**, 45, 110-125.
303. Lei, X.M.; D.M. Siringoringo; Z. Sun, and Y. Fujino. Displacement response estimation of a cable-stayed bridge subjected to various loading conditions with one-dimensional residual convolutional autoencoder method. Structural Health Monitoring-an International Journal **2022**, 17.
304. Park, H.S.; T. Hong; D.E. Lee; B.K. Oh, and B. Glisic. Long-term structural response prediction models for concrete structures using weather data, fiber-optic sensing, and convolutional neural network. Expert Systems with Applications **2022**, 201, 12.
305. Du, B.W.; C.M. Lin; L.L. Sun; Y.P. Zhao, and L.C. Li. Response Prediction Based on Temporal and Spatial Deep Learning Model for Intelligent Structural Health Monitoring. Ieee Internet of Things Journal **2022**, 9, 13364-13375.
306. Wang, Z.W.; A.D. Li; W.M. Zhang, and Y.F. Zhang. Long-term missing wind data recovery using free access databases and deep learning for bridge health monitoring. Journal of Wind Engineering and Industrial Aerodynamics **2022**, 230, 14.
307. Hareendran, S.P. and A. Alipour. Prediction of nonlinear structural response under wind loads using deep learning techniques. Applied Soft Computing **2022**, 129, 13.
308. Jung, K.C. and S.H. Chang. Advanced deep learning model-based impact characterization method for composite laminates. Composites Science and Technology **2021**, 207, 10.
309. Kumar, A.; S. Singla; A. Kumar; A. Bansal, and A. Kaur. Efficient Prediction of Bridge Conditions Using Modified Convolutional Neural Network. Wireless Personal Communications **2022**, 125, 29-43.
310. Choi, Y.; J. Lee, and J. Kong. Performance Degradation Model for Concrete Deck of Bridge Using Pseudo-LSTM. Sustainability **2020**, 12, 19.
311. Lee, I.Y.; J. Jang, and Y.B. Park. Advanced structural health monitoring in carbon fiber-reinforced plastic using real-time self-sensing data and convolutional neural network architectures. Materials & Design **2022**, 224, 13.
312. Tang, Q.Z.; J.Z. Xin; Y. Jiang, et al. Novel identification technique of moving loads using the random response power spectral density and deep transfer learning. Measurement **2022**, 195, 14.
313. Ozelim, L.; L.P.D. Borges; A.L.B. Cavalcante, et al. Structural Health Monitoring of Dams Based on Acoustic Monitoring, Deep Neural Networks, Fuzzy Logic and a CUSUM Control Algorithm. Sensors **2022**, 22, 25.

314. Chen, L.; A. Gallet; S.S. Huang; D. Liu, and D. Smyl. Probabilistic cracking prediction via deep learned electrical tomography. Structural Health Monitoring-an International Journal **2022**, 21, 1574-1589.
